# Interaction of Macromolecular Chain with Phospholipid Membranes in Solutions: A Dissipative Particle Dynamics Simulation Study

**DOI:** 10.3390/molecules28155790

**Published:** 2023-07-31

**Authors:** Yuane Wang, Xuankang Mou, Yongyun Ji, Fan Pan, Shiben Li

**Affiliations:** 1 Department of Physics, Wenzhou University, Wenzhou 325035, China; 21451026015@stu.wzu.edu.cn (Y.W.); 19211021126@stu.wzu.edu.cn (X.M.); yyji@wzu.edu.cn (Y.J.); 2 School of Data Science and Artificial Intelligence, Wenzhou University of Technology, Wenzhou 325035, China

**Keywords:** macromolecular chain, phospholipid membrane, pulling force, dynamic process

## Abstract

The interaction between macromolecular chains and phospholipid membranes in aqueous solution was investigated using dissipative particle dynamics simulations. Two cases were considered, one in which the macromolecular chains were pulled along parallel to the membrane surfaces and another in which they were pulled vertical to the membrane surfaces. Several parameters, including the radius of gyration, shape factor, particle number, and order parameter, were used to investigate the interaction mechanisms during the dynamics processes by adjusting the pulling force strength of the chains. In both cases, the results showed that the macromolecular chains undergo conformational transitions from a coiled to a rod-like structure. Furthermore, the simulations revealed that the membranes can be damaged and repaired during the dynamic processes. The role of the pulling forces and the adsorption interactions between the chains and membranes differed in the parallel and perpendicular pulling cases. These findings contribute to our understanding of the interaction mechanisms between macromolecules and membranes, and they may have potential applications in biology and medicine.

## 1. Introduction

Phospholipid membranes are semipermeable structures that serve as the primary components of cell membranes, separating the intracellular and extracellular environments and regulating the exchange of substances [1,2]. Interactions between phospholipid membranes and macromolecular chains (i.e., polymer chains), particularly biopolymers, such as lipid-tethered DNA, are common and inevitable [3]. Thus, the investigation of these interactions has garnered much attention in the biomedical field, driving advancements in medical carriers [4,5,6].

Recent years have witnessed numerous studies focusing on the interaction of macromolecules with phospholipid membranes through experimental approaches [6,7,8,9,10,11,12,13,14,15,16,17,18,19,20,21,22,23,24]. For example, Brodszkij et al. investigated the interaction between two acrylate macromolecules with carboxylic acid side groups and phospholipid membranes during drug delivery [16]. Their findings revealed a conformation transition from a coiled to a spherical structure for the macromolecules poly(carboxyethyl acrylate) (PCEA) and poly(carboxypentyl acrylate) (PCPA) when the pH was reduced from 7 to 4. Additionally, a pH-triggered partially reversible aggregate with giant unilamellar vesicles was observed. The authors suggested that PCEA and PCPA can passively diffuse across cell membranes, highlighting their potential in the medical field [16]. Compared with simple macromolecules, such as homopolymers, the interaction between block copolymers and phospholipid membranes exhibits unique properties due to the different chemical properties of the constituent blocks [17,18,19,22]. For example, Van Zee et al. investigated the interaction of poloxamer and its analogs, which comprise different blocks, with phospholipid membranes [22]. The experimental results showed that the poly(butylene oxide) (PBO)-poly(ethylene oxide) (PEO) diblock polymers exhibited remarkably stronger binding than analogous copolymers containing polyphenylene oxide (PPO). Recently, Ge et al. used a photovoltage transient method combined with atomic force microscopy and other techniques to monitor real-time interaction processes between polymers and phospholipids [23]. In their study, three typical polymers, polyoxyethylene (35), lauryl ether (Brij35), and polyvinylpyrrolidone (PVPK30), were used to interact with phospholipid membranes under low ionic strength and neutral pH conditions. The authors explained the toxicity and low toxicity of polymer–phospholipid membrane interactions in living cells based on the charge relaxation time constant of bilayer discharge at the membrane–solution interface, emphasizing the enhanced effects of polymer surfactants and membrane interactions [23].

Various computer simulations have been conducted employing all-atom or coarse-grained (CG) models to gain a better understanding of experimental results regarding the interaction of macromolecules with phospholipid membranes. These simulations encompassed a range of polymers, including biomacromolecules and block copolymers [3,25,26,27,28,29,30,31,32,33,34,35]. For example, Beldowski et al. used all-atom molecular dynamics simulations to investigate the interactions between hyaluronan acid and dipalmitoylphosphatidylcholine membranes [31]. The results highlighted the crucial roles played by hydrogen bonds and hydrophobic contacts in bridging the carboxylate group of hyaluronic acid and the phosphate group of phospholipid molecules. Other macromolecules, such as DNA and antimicrobial peptides, were also selected to investigate their interactions with membranes [3,33]. Hezaveh et al. used CG molecular dynamics simulations to investigate the interactions between triblock polymers and membranes, observing the percolation of block polymers through membranes when the length of the PPO block was comparable to the membrane thickness [25]. The results indicated that polymers with a long hydrophobic middle block can deeply penetrate the hydrocarbon core of the membrane, whereas shorter hydrophobic blocks exhibit weaker binding, aligning with experimental findings and explicable through system free energies [7,9,10]. Rabbel et al. used the Monte Carlo simulation method to investigate the interactions of amphiphilic ABA triblock copolymers with phospholipid membranes, indicating that the hydrophobic blocks have different effects in the transmembrane state and harpin state [27]. Nawaz et al. performed a comprehensive study combining experiments and computer simulations, suggesting that the hydrophilic blocks in poly(ethylene oxide)-poly(propylene oxide)-poly(ethylene oxide) (PEO-PPO-PEO)triblock copolymers interact with the polar head groups of lipid molecules, leading to membrane structure modification [26]. The experimental observations are in good agreement with the computational predictions. Furthermore, Houang et al. used all-atom molecular dynamics simulations to explore the interactions between PEO-PPO-PEO triblock copolymers and POPC membranes [28]. The results showed that the triblock copolymer P188 only inserts into low-density or damaged areas of the membrane, which strongly depend on its overall hydrophobicity and are consistent with experimental observations [7,8]. Most of these studies focused on the interaction between block copolymers and lipid membranes in the absence of external pulling forces. Dai et al. used dissipative particle dynamics (DPD) simulations to investigate the interaction of bottlebrush copolymers with phospholipid membranes in the presence of external pulling force, and the results showed that the hydrophilic head of the brush molecules acting on the surface of phospholipid membranes produces different morphology changes under the external pulling force along membrane surfaces, and the phenomenon of phospholipid membrane rupture can be clearly observed [35]. However, the interaction between macromolecules and lipid membranes under pulling forces has received limited attention, and exploring the mechanisms underlying such interactions is still valuable.

In this study, we used DPD simulations to investigate the interaction mechanism between macromolecules and phospholipid membranes under the influence of pulling forces of varying strengths and varying directions. We selected linear polymer chains as model macromolecules and considered two cases: pulling forces parallel and perpendicular to the membrane surfaces. We specifically examined changes in the shapes of polymer chains and the structures of membranes by adjusting the strength of the pulling forces in both cases. The subsequent section introduces the DPD method and the model used. Section 3 provides a detailed discussion of the results. Section 4 summarizes the conclusions.

## 2. Results

**Figure 1:**
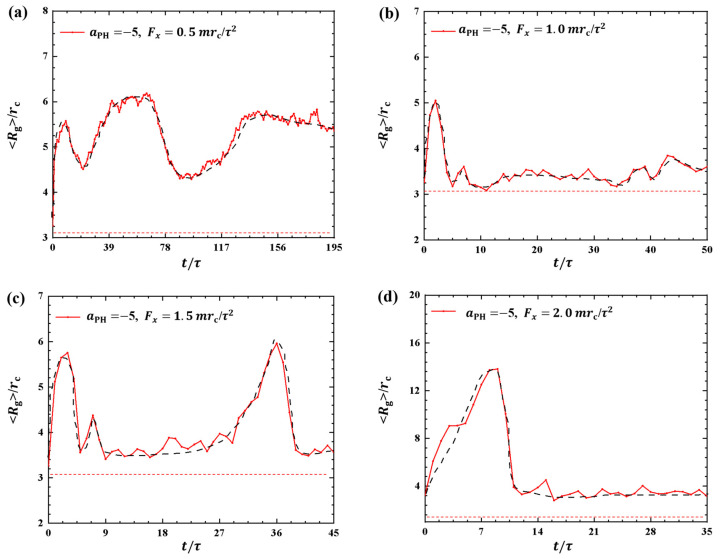
Variation of gyration radius $\langle R_{g}\rangle$ for the polymer chains in the weak adsorption cases of $a_{{}_{\mathrm{PH}}}=-5$. The pulling forces applied to the polymer chain are along the *x* direction as (**a**) $F_{x}=0.5\;mr_{c}/\tau^{2}$, (**b**) $F_{x}=1.0\;mr_{c}/\tau^{2}$, (**c**) $F_{x}=1.5\;mr_{c}/\tau^{2}$, and (**d**) $F_{x}=2.0\;mr_{c}/\tau^{2}$. The red dotted lines denote the values of gyration radius for the polymer with coil conformation. The black dashed lines show the variation trends.

**Figure 2:**
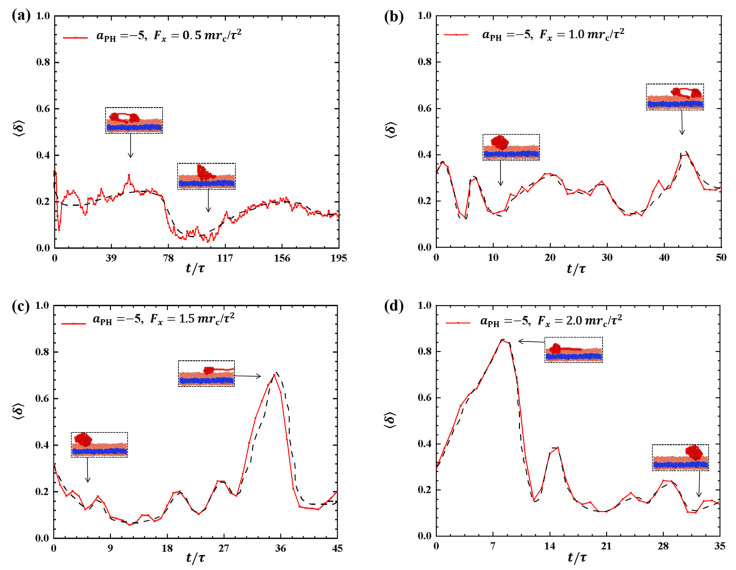
Variation of shape factor $\langle \delta \rangle$ for the polymer chains in the weak adsorption cases of $a_{{}_{\mathrm{PH}}}=-5$. The pulling force applied to the polymer chain is along the *x* direction as (**a**) $F_{x}=0.5\;mr_{c}/\tau^{2}$, (**b**) $F_{x}=1.0\;mr_{c}/\tau^{2}$, (**c**) $F_{x}=1.5\;mr_{c}/\tau^{2}$, and (**d**) $F_{x}=2.0\;mr_{c}/\tau^{2}$. The black dashed lines show the variation trends. The typical conformations are also inserted.

**Figure 3:**
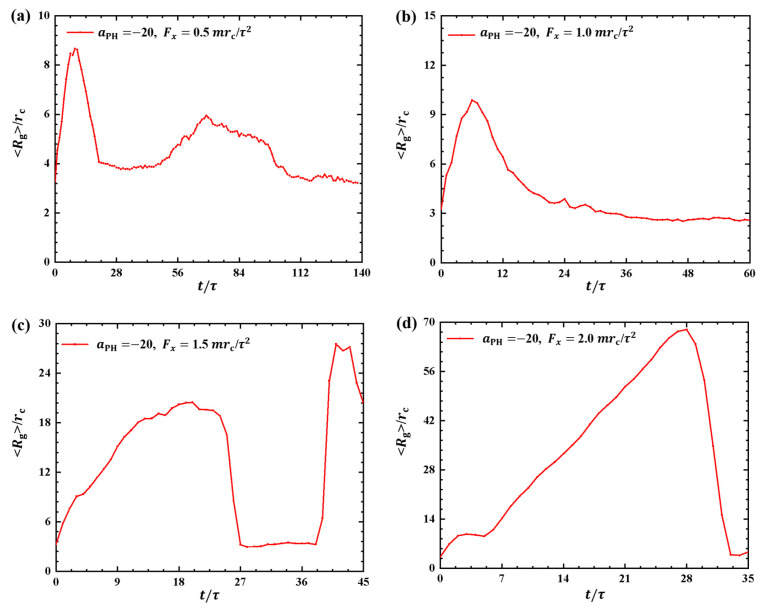
Variation of gyration radius $\langle R_{g}\rangle$ for the polymer chains in the strong adsorption cases of $a_{{}_{\mathrm{PH}}}=-20$. The pulling force applied to the polymer chain is along the *x* direction as (**a**) $F_{x}=0.5\;mr_{c}/\tau^{2}$, (**b**) $F_{x}=1.0\;mr_{c}/\tau^{2}$, (**c**) $F_{x}=1.5\;mr_{c}/\tau^{2}$ and (**d**) $F_{x}=2.0\;mr_{c}/\tau^{2}$.

**Figure 4:**
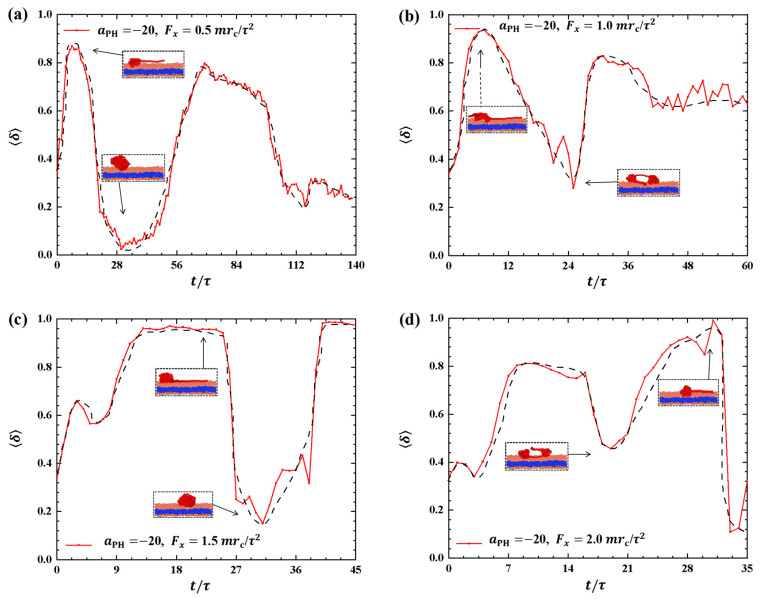
Variation of shape factor $\langle \delta \rangle$ for the polymer chains in the strong adsorption cases of $a_{{}_{\mathrm{PH}}}=-20$. The pulling force applied to the polymer chain is along the *x* direction as (**a**) $F_{x}=0.5\;mr_{c}/\tau^{2}$, (**b**) $F_{x}=1.0\;mr_{c}/\tau^{2}$, (**c**) $F_{x}=1.5\;mr_{c}/\tau^{2}$, and (**d**) $F_{x}=2.0\;mr_{c}/\tau^{2}$. The black dashed lines show the variation trends. The typical conformations are also inserted.

**Figure 5:**
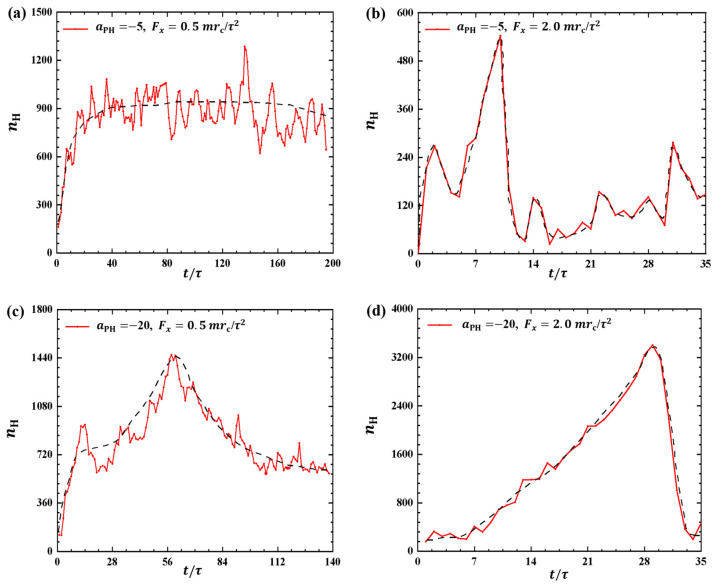
The number of hydrophilic phospholipid molecules that lie in the range $z=10\;r_{c}-15\;r_{c}$ as functions of the pulling time in the cases of (**a**) weak adsorption $a_{{}_{\mathrm{PH}}}=-5$ and weak pulling force $F_{x}=0.5\;mr_{c}/\tau^{2}$, (**b**) weak adsorption $a_{{}_{\mathrm{PH}}}=-5$ and strong pulling force $F_{x}=2.0\;mr_{c}/\tau^{2}$, (**c**) strong adsorption $a_{{}_{\mathrm{PH}}}=-20$ and weak pulling force $F_{x}=0.5\;mr_{c}/\tau^{2}$, and (**d**) strong adsorption $a_{{}_{\mathrm{PH}}}=-20$ and strong pulling force $F_{x}=2.0\;mr_{c}/\tau^{2}$. The black dashed lines show the variation trends.

**Figure 6:**
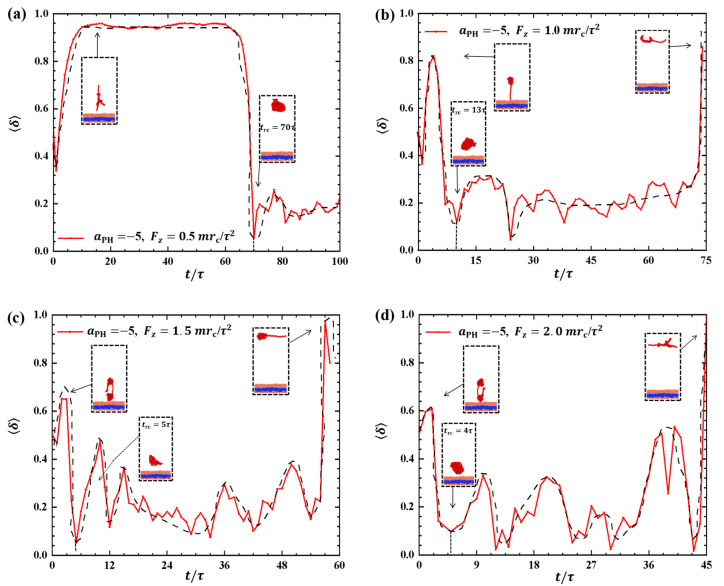
Variation of shape factor $\langle \delta \rangle$ for the polymer chains in the weak adsorption cases of $a_{{}_{\mathrm{PH}}}=-5$. The pulling force applied to the polymer chain is along the *z* direction as (**a**) $F_{z}=0.5\;mr_{c}/\tau^{2}$, (**b**) $F_{z}=1.0\;mr_{c}/\tau^{2}$, (**c**) $F_{z}=1.5\;mr_{c}/\tau^{2}$, and (**d**) $F_{z}=2.0\;mr_{c}/\tau^{2}$. The black dashed lines show the variation trends. The typical conformations are also inserted.

**Figure 7:**
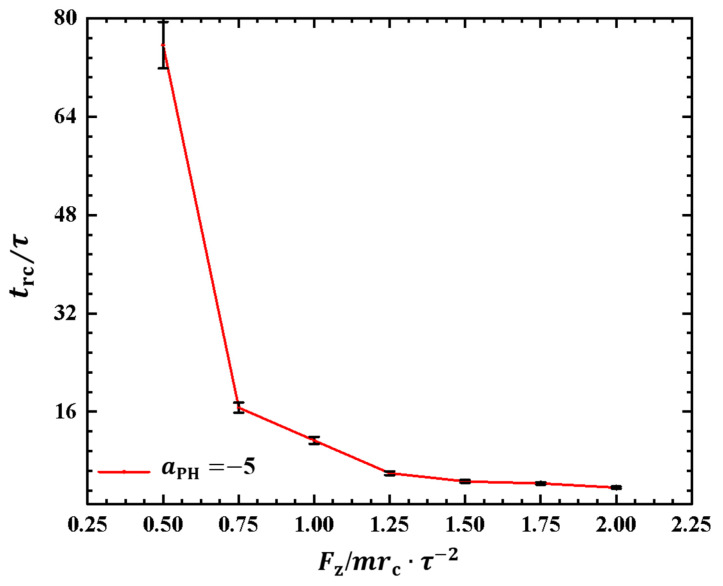
The rod–coil transition period as a function of pulling force applied along the *z* direction in the weak adsorption cases of $a_{{}_{\mathrm{PH}}}=-5$. The error bars are also shown.

**Figure 8:**
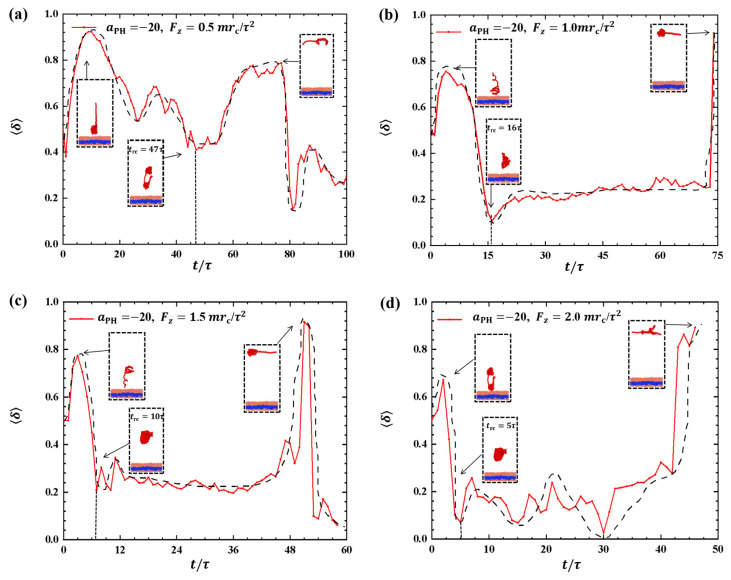
Variation of shape factor $\langle \delta \rangle$ for the polymer chains in the strong adsorption cases of $a_{{}_{\mathrm{PH}}}=-20$. The pulling force applied to the polymer chain is along the *z* direction as (**a**) $F_{z}=0.5\;mr_{c}/\tau^{2}$, (**b**) $F_{z}=1.0\;mr_{c}/\tau^{2}$, (**c**) $F_{z}=1.5\;mr_{c}/\tau^{2}$, and (**d**) $F_{z}=2.0\;mr_{c}/\tau^{2}$. The black dashed lines show the variation trends. The typical conformations are also inserted.

**Figure 9:**
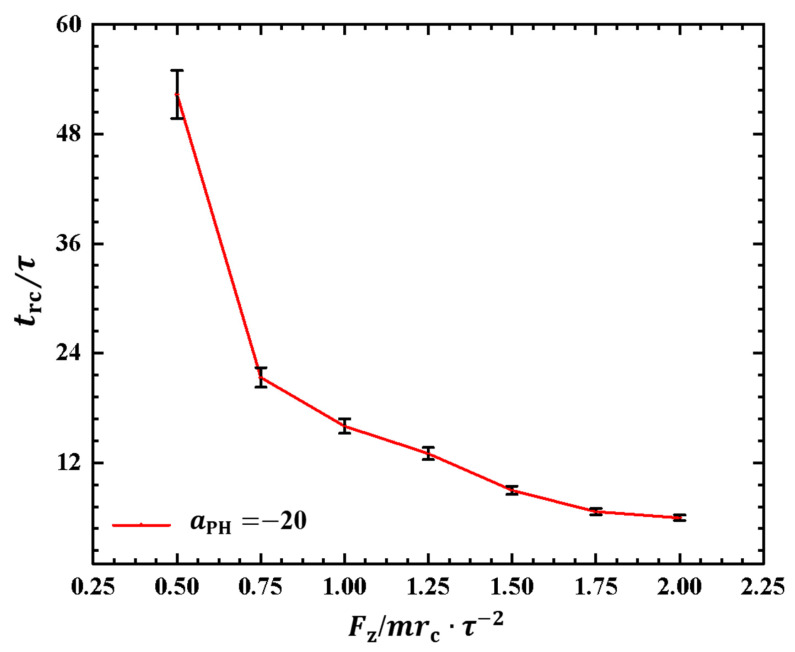
The rod–coil transition period as a function of pulling force applied along the *z* direction in the strong adsorption cases of $a_{{}_{\mathrm{PH}}}=-20$. The error bars are also shown.

**Figure 10:**
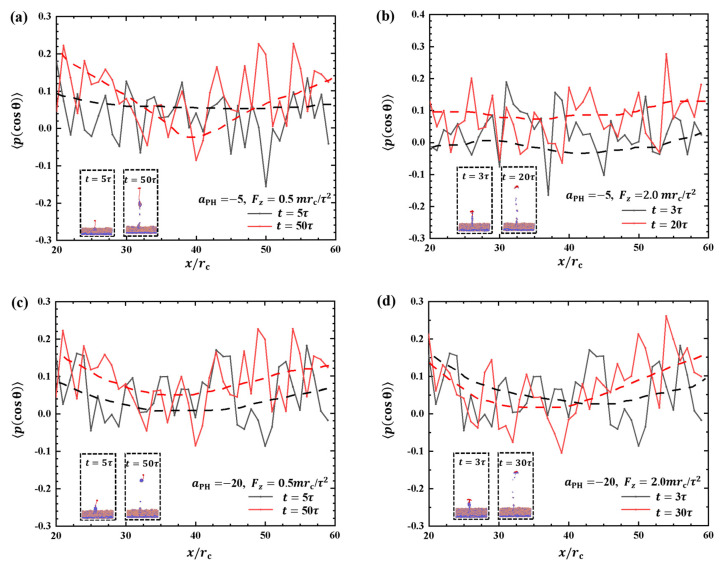
Order parameter profiles of phospholipid films in the cases of (**a**) weak adsorption $a_{{}_{\mathrm{PH}}}=-5$ and weak pulling force $F_{z}=0.5\;mr_{c}/\tau^{2}$, (**b**) weak adsorption $a_{{}_{\mathrm{PH}}}=-5$ and strong pulling force $F_{z}=2.0\;mr_{c}/\tau^{2}$, (**c**) strong adsorption $a_{{}_{\mathrm{PH}}}=-20$ and weak pulling force $F_{z}=0.5\;mr_{c}/\tau^{2}$, and (**d**) strong adsorption $a_{{}_{\mathrm{PH}}}=-20$ and strong pulling force $F_{z}=2.0\;mr_{c}/\tau^{2}$. The black curve corresponds to the moment when the polymer chain is about to separate from the phospholipid membrane, and the red curve corresponds to the moment when the phospholipid membrane changes more significantly at the end of the action.

In the current simulations, we investigated the effects of pulling forces along the *x* and *z* directions (denoted as $F_{x}$ and $F_{z}$) on the conformation of polymer chains, considering both weak and strong adsorption interactions between the polymer chains and membranes ($a_{{}_{\mathrm{PH}}}=-20$ and $a_{{}_{\mathrm{PH}}}=-5$). We focused on a single pulling process where the polymers were pulled from the left to right side of simulation box by $F_{x}$ or from the bottom to top side of box by $F_{z}$. The results are discussed in terms of the gyration radius, shape factor, order parameter, and bead density in the parallel (Figures 1–5) and perpendicular pulling case (Figures 6–10). In both cases, the strengths of the pulling forces were set to be $F_{x,z}=$ 0.5, 1.0, 1.5, and 2.0, in the unit of $mr_{c}/\tau^{2}$. It turns out that different initial random conformations of polymer chains have little effects on the pulling processes. This enables us to describe the dynamics processes using only one replication because the results are reproducible by using the random inputting. Therefore, we used one replica for each set of parameters, where the variation trends were labeled by the dashed lines in the dynamics processes (See more replicas in Supplementary Materials).

### 2.1. Pulling Forces along the Direction Parallel to Membrane Surfaces

#### 2.1.1. Conformations of Polymer Chains under Weak Adsorption

We first examined the conformations of polymer chains under weak adsorption ($a_{{}_{\mathrm{PH}}}=-5$) when the pulling forces were parallel to the membrane surfaces. The gyration radius, which describes the size of the polymer chain, was evaluated using the mean square radius of gyration $\langle R_{g}^{2}\rangle$ given by Equation (1) [36]

(1) $$\langle R_{g}^{2}\rangle =\frac{1}{N}\sum_{i=1}^{N}\langle {\left(r_{i}-r_{c}\right)}^{2}\rangle ,$$

where sum covers all beads and $r_{c}$ denotes the mass center of the polymer chain. Figure 1 shows the gyration radius of the polymers during the dynamics processes under various strengths of pulling forces, namely, $F_{x}=0.5\;mr_{c}/\tau^{2},1.0\;mr_{c}/\tau^{2},1.5\;mr_{c}/\tau^{2}$, and $2.0\;mr_{c}/\tau^{2}$. The pulling time *t* is not the same for different pulling forces, and as expected, a larger pulling force leads to a shorter pulling time required to complete a single process. The results indicate t=195τ,50τ,45τ and 35τ under $F_{x}=0.5\;mr_{c}/\tau^{2},1.0\;mr_{c}/\tau^{2},1.5\;mr_{c}/\tau^{2}$, and $2.0\;mr_{c}/\tau^{2}$, as shown in Figure 1a–d, respectively. When the pulling force $F_{x}$ is small, the pulling time *t* is relatively long. As $F_{x}$ reaches a certain magnitude, *t* changes more slowly. Another interesting observation is the presence of multiple relaxation processes in the case of $F_{x}=0.5\;mr_{c}/\tau^{2}$ case, where the gyration radius undergoes three distinct changes over from t=0 to t=195τ. This behavior may explain the slower movement of the polymer chain under small pulling forces as relaxation processes have been observed in other types of macromolecules during dynamic processes [35].

In this study, we observed similar relaxation processes when typical linear chains interacted with phospholipid membranes. Here, it is difficult for the polymer chains to reach a sort of stationary state. One reason is that the forces are always applied to the polymer chain during the whole pulling process, and the polymer chain is always moving with the external forces, so the polymer chain is always in non-equilibrium within the range of our observations. Another reason is that the pulling time is not long enough in the current simulations. It is reasonable to suspect that if the pulling time is long enough, it may eventually reach an equilibrium state. Due to computational limitations, we are unable to observe such equilibrium states in the current simulations. However, we can assume an ideal equilibrium where the polymer chain exhibits a random coil conformation with gyration radius of $\langle R_{g}\rangle =3.2\;r_{c}$, labeled as red dotted lines in Figure 1. The comparisons clearly showed the variations of $\langle R_{g}\rangle$ from the equilibrium states in the dynamics processes.

To provide a more detailed description of the shape of macromolecules, we also analyzed the shape factor, which characterizes the shape of the polymer chain. The shape factor can be expressed as [37,38]

(2) $$\langle \delta \rangle =1-3\langle \frac{L_{1}^{2}L_{2}^{2}+L_{2}^{2}L_{2}^{2}+L_{1}^{2}L_{3}^{2}}{\left(L_{1}^{2}+L_{2}^{2}+L_{3}^{2}\right)}\rangle \;,\;$$

where $L_{1}^{2},L_{2}^{2}$, and $L_{3}^{2}$ represent the three eigenvalues of the gyration radius tensor $R_{g}^{2}$. The tensor $R_{g}^{2}$ is defined by Equation (2), and its elements $R_{g\alpha \beta }^{2}$ are given by equation

(3) $$R_{g}^{2}=\left(\begin{array}{ccc} R_{gxx}^{2} & R_{gxy}^{2} & R_{gxz}^{2}\\ R_{gyx}^{2} & R_{gyy}^{2} & R_{gyz}^{2}\\ R_{gzx}^{2} & R_{gzy}^{2} & R_{gzz}^{2} \end{array}\right),$$

(4) $$\langle R_{g\alpha \beta }^{2}\rangle =\frac{1}{N}\sum_{i=1}^{N}\langle \left(r_{i,\alpha }-r_{c,\alpha }\right)\left(r_{i,\beta }-r_{c,\beta }\right)\rangle ,\alpha ,\beta \in \left\{x,y,z\right\},$$

where *N* represents the number of beads, and $r_{i,x}$ in brackets denotes the *x* coordinate of the *i*-th bead. The shape factor $\langle \delta \rangle$ provides a straightforward description of the conformations of polymer chains. The conformation of the chain tends to be spherical when $\langle \delta \rangle$ = 0. The chain exhibits a circular structure when $\langle \delta \rangle$ = 0.5. The conformation of the chain resembles a rod-like structure when $\langle \delta \rangle$ = 1.0 [38].

To provide a clearer description of the conformational changes in the linear polymer chain during the dynamic processes, we examined the shape factors for polymer chains with weak adsorptions, as shown in Figure 2. The results showed distinct variations in the shape factors under different pulling forces, as shown in Figure 2a–d, where the conformations are also illustrated. In Figure 2a,b, the shape factors $\langle \delta \rangle$ range between 0 and 0.5 when the pulling forces are $F_{x}=0.5\;mr_{c}/\tau^{2}$ and $1.0\;mr_{c}/\tau^{2}$, suggesting that the conformations of the polymer chains oscillate between spherical and circular structures, as indicated in the inset. As the pulling force is increased to $1.5\;mr_{c}/\tau^{2}$, as shown in Figure 2c, the conformation of the polymer chain changes significantly, with $\langle \delta \rangle$ increasing from 0.14 to 0.71 due to the stronger pulling force and then decreasing to about 0.2, i.e., the polymer chain transits from a sphere to a near-rod shape and then relaxes back to a sphere again. The experiment reported that the PCEA and PCPA macromolecules undergo the similar coil–rod conformational transitions when these macromolecules interact with the phospholipid membranes at different pH values [16]. Here, we observed that the coil–rod conformational transition induced the pulling forces. A similar transition occurs under the pulling force $F_{x}=2.0\;mr_{c}/\tau^{2}$, as shown in Figure 2d. However, the period of the sphere-rod transition is shorter than that observed in the $F_{x}=2.0\;mr_{c}/\tau^{2}$ case due to the larger pulling forces. The spring model employed in previous studies can provide an explanation for the observed polymer conformations [35,39]. Our observations align with the findings from previous simulations, where the conformational changes of polymer chains were described using shape factors under varying pulling forces. Our results about the shape factors also showed that the polymers have deviations from the ideal coil conformations with $\langle \delta \rangle =0$, where the polymers can be assumed in equilibrium states.

#### 2.1.2. Conformations of Polymer Chains under Strong Adsorption

We investigated the conformational behavior of polymer chains in dynamic processes when the adsorption interactions between the membrane surfaces and polymer chains are strong.

Figure 3 and Figure 4 illustrate the results, where the adsorption interactions are set to $a_{{}_{\mathrm{PH}}}=-20$, the pulling forces are set to $F_{x}=0.5\;mr_{c}/\tau^{2},1.0\;mr_{c}/\tau^{2},1.5\;mr_{c}/\tau^{2}$, and $2.0\;mr_{c}/\tau^{2}$ for convenient comparison with the weak adsorption cases. The corresponding pulling times are t=140τ, 60τ, 45τ, and 35τ under $F_{x}=0.5\;mr_{c}/\tau^{2},\;1.0\;mr_{c}/\tau^{2},\;1.5\;mr_{c}/\tau^{2}$, and $2.0\;mr_{c}/\tau^{2}$, as shown in Figure 3a–d. Similar to the weak adsorption cases, the pulling time *t* decreases gradually when the pulling force increases to $1.0\;mr_{c}/\tau^{2}$. However, the gyration radius exhibits remarkable variation under strong adsorption from the membrane surfaces. For example, the gyration radius of the polymer chain reaches a local maximum peak of $8.67\;r_{c}$ at t=9τ and gradually returns to equilibrium (Figure 3a). When the pulling force is $F_{x}=1.5\;mr_{c}/\tau^{2}$, the polymer chain displays noticeable oscillatory behavior, reaching a maximum of $\langle R_{g}\rangle =27.7\;r_{c}$ at t=38τ. This oscillation is similar to the case with $F_{x}=0.5\;mr_{c}/\tau^{2}$ shown in Figure 3a. However, the polymer chains undergo a single and more pronounced oscillation when $F_{x}=1.0\;mr_{c}/\tau^{2}$ and $2.0\;mr_{c}/\tau^{2}$, as shown in Figure 3b,d. In particular, the gyration radius of the polymer chain can reach a maximum value of $67.9\;r_{c}$ when $F_{x}=2.0\;mr_{c}/\tau^{2}$. Therefore, we can conclude that the pulling forces easily alter the polymer size due to the strong adsorption between the polymer and membrane. Furthermore, the results also indicated that there are more obvious deviations of polymer conformations from the equilibrium in the strong adsorption cases than those observed in the weak adsoprtion cases. In the single-molecule experiments and simulations, the similar periodic motion has been observed in quantities, such as polymer orientation, revealing characteristic periodicity in flow [40]. Here, we observed both periodic and nonperiodic transitions in polymer chains under strong adsorption. Furthermore, we noticed remarkable conformational variations in the linear polymer chains compared with polymer brushes observed in previous work [35]. This discrepancy arises because brushes have the potential for stronger repulsive interactions, leading to more pronounced conformational transitions in linear chains [41].

Discussing the shape factors of polymer chains under strong adsorption is important, as shown in Figure 4. The shape factor is presented as a function of time *t* during the pulling processes under different pulling forces: $F_{x}=0.5\;mr_{c}/\tau^{2},1.0\;mr_{c}/\tau^{2},1.5\;mr_{c}/\tau^{2}$, and $2.0\;mr_{c}/\tau^{2}$ in Figure 4a–d, respectively. The results reveal remarkable changes in the shape of polymer chains under strong adsorption, where the shape factor varies between 0 and 1. In particular, the maximum values of $\langle \delta \rangle$ are 0.87, 0.94, 0.97, and 1.0 when $F_{x}=0.5\;mr_{c}/\tau^{2},\;1.0\;mr_{c}/\tau^{2},\;1.5\;mr_{c}/\tau^{2}$, and $2.0\;mr_{c}/\tau^{2}$, respectively. This indicates that polymer chains tend to adopt a rod-like shape under strong pulling forces, which obviously deviates from the equilibrium coil conformation in most pulling processes. The shape factor exhibits similar oscillatory periods compared with the corresponding changes in the gyration radius when $F_{x}=0.5\;mr_{c}/\tau^{2}$ and $1.5\;mr_{c}/\tau^{2}$. However, clear differences are observed between the shape factors and gyration radius when $F_{x}=1.0\;mr_{c}/\tau^{2}$ and $2.0\;mr_{c}/\tau^{2}$, as shown in Figure 4b,d. Specifically, we observed periodic oscillations in the shape factors but not in the gyration radius when $F_{x}=1.0\;mr_{c}/\tau^{2}$ and $2.0\;mr_{c}/\tau^{2}$. Moreover, the oscillation periods are longer under strong adsorption than under weak adsorption due to the interaction between polymer chains and the membrane. This can be explained by the spring model, where the spring takes more time to relax. Additionally, the greater the pulling force, the greater the fluctuation of $\langle \delta \rangle$, indicating that increasing the pulling force enhances the conformational changes of the polymer chain in the phospholipid film. Furthermore, the shape factor of the polymer chains tends to increase and then decrease under strong adsorption when the pulling forces are the same.

#### 2.1.3. Variance of Bead Number in Membrane

In the pulling processes, the pulling forces and adsorption interactions between the polymer and membrane not only affect the conformation of the polymer chain but also can affect the membranes. To investigate the effect of pulling forces on phospholipid membranes, we examine the bead number of phospholipid molecules in the dynamic processes. Figure 5 shows the plot of $n_{{}_{H}}$ as a function of pulling time *t*, where $n_{{}_{H}}$ represents the number of hydrophilic head beads of phospholipid molecules located within the range of $z=10\;r_{c}-15\;r_{c}$. This range allows us to observe the detachment of phospholipid molecules from the membrane surfaces since the phospholipid molecules initially reside within the range of $z=1\;r_{c}-9\;r_{c}$ in the equilibrium state, where the total number of head beads in the upper layer $n_{{}_{0}H}$ is about 79,753.

For weak adsorption $a_{{}_{\mathrm{PH}}}=-5$, we investigated the effect of polymer chains on phospholipid membranes under two different external pulling forces ($F_{x}=0.5\;mr_{c}/\tau^{2}$ and $2.0\;mr_{c}/\tau^{2}$), as shown in Figure 5a,b. Specifically, the bead number $n_{{}_{H}}$ increases and subsequently maintains an approximately constant value of $n_{{}_{H}}=900$ under the small pulling force $F_{x}=0.5\;mr_{c}/\tau^{2}$, as shown in Figure 5a. However, for strong pulling forces, such as $F_{x}=2.0\;mr_{c}/\tau^{2}$ (Figure 5b), the bead number $n_{H}$ increases rapidly to a maximum value of $n_{{}_{H}}=540$ and then quickly decreases to a low value of $n_{{}_{H}}=120$. This indicates that more lipid molecules detach from the membrane surfaces under the smaller pulling force compared with the stronger pulling forces. Moreover, the results indicate that phospholipid membranes can repair quickly under strong pulling forces, where the membrane can be regarded as equilibrium conformation when $n_{{}_{H}}=0$. This observation suggests that polymer chains under a smaller pulling force interact better with phospholipid membranes than those under a larger pulling force, which agrees with our previous results [35]. Naturally, the damage to the membranes is also influenced by the adsorption interactions between the polymer chains and membranes. Next, we considered the strong adsorption cases, as shown in Figure 5c,d, where $a_{{}_{\mathrm{PH}}}=-20$ for two different external pulling forces ($F_{x}=0.5\;mr_{c}/\tau^{2}$ and $2.0\;mr_{c}/\tau^{2}$). Compared with the weak adsorption cases, more molecules can detach from the membrane surfaces due to the strong interactions between the polymers and membranes. In particular, the bead number $n_{{}_{H}}$ increases to $n_{{}_{H}}=1440$ and then decreases to $n_{{}_{H}}=570$ at $F_{x}=0.5\;mr_{c}/\tau^{2}$. For the strong pulling force, we observed that the bead number $n_{{}_{H}}$ can even increase to $n_{{}_{H}}=3400$ and then decrease to $n_{{}_{H}}=480$, namely, the number of head particles $n_{{}_{H}}$ decreases from 4.2 percent to 0.6 percent of the total number of head particles in the upper layer $n_{{}_{0}H}$. This suggests that phospholipid membranes suffer remarkable damage under strong pulling forces when the adsorption between the polymer and membrane is also strong. Bead numbers have also been used to investigate other polymer features where the coarse-grained and all-atom molecular dynamics simulations were employed [25,31,34]. In this case, we used them to describe the membrane’s response caused by the pulling forces applied to the linear polymer chains. Previous work suggested that the effect of linear chain polymers on the dielectric film is less pronounced than with other types of polymers [41].

### 2.2. Pulling Forces Perpendicular to Membrane Surfaces

#### 2.2.1. Conformations of Polymer Chains under Weak Adsorption

In this subsection, we examine cases where the polymer chains are pulled from the membrane surface in a direction perpendicular to the membrane surfaces, considering both weak and strong adsorptions ($a_{{}_{\mathrm{PH}}}=-5$ and −20, respectively). We plotted the shape factor $\langle \delta \rangle$ as a function of pulling time *t* under various pulling forces when $a_{{}_{\mathrm{PH}}}=-5$, as shown in Figure 6. Under weak pulling forces ($F_{z}=0.5\;mr_{c}/\tau^{2}$), the shape factor $\langle \delta \rangle$ exhibits large fluctuation amplitude initially. Subsequently, $\langle \delta \rangle$ gradually tends to a steady state, and at $t_{\mathrm{rc}}=70\;\tau$, $\langle \delta \rangle$ drops sharply to its minimum value, as shown in Figure 6a. This indicated that the polymer conformation obviously deviates from the coil equilibrium state when the small pulling forces are applied. The polymer chain maintains a rod-like shape for an extended period due to slow detachment of the polymer chain from the membrane surface under weak pulling forces. However, the interactions between the membrane and polymer chains cease because the polymers are completely pulled away from the membrane surfaces, resulting in the polymer chain adopting a coil conformation. A similar rod–coil transition has been observed in simulations investigating interactions between polymer chains and membranes without pulling forces [25]. In this case, we observed rod–coil conformational transitions under the influence of pulling forces. For higher pulling forces, we noticed increased fluctuation in the shape factor, with the oscillation frequency of the shape factor increasing as the pulling force increased to $F_{z}=1.0\;mr_{c}/\tau^{2},1.5\;mr_{c}/\tau^{2}$, and $2.0\;mr_{c}/\tau^{2}$, as shown in Figure 6b–d. For example, the polymer chain transitions into the nearly rod-like conformation for the first time at approximately t=10.0τ,3.8τ,3.0τ, and 2.0τ when $F_{z}=1.0\;mr_{c}/\tau^{2},1.5\;mr_{c}/\tau^{2}$, and $2.0\;mr_{c}/\tau^{2}$, respectively. This indicates that the polymer chain can quickly transition into rod-like conformations under strong pulling forces, which aligns with previous findings [42]. The shape factor exhibits fluctuations as the polymer chains are pulled away from the membranes, similar to previous observations [35]. The pulling time *t* within the simulation boxes decreases as the pulling force increases, with t=100τ, 75τ, and 60τ when $F_{z}=1.0\;mr_{c}/\tau^{2},\;1.5\;mr_{c}/\tau^{2}$, and $2.0\;mr_{c}/\tau^{2}$, respectively.

As depicted in Figure 6, the polymer chain exhibits oscillatory behavior under the pulling force perpendicular to the membrane surface. Investigating the period of the rod–coil conformational transition for the first time is valuable. We plotted the rod–coil transition periods $t_{\mathrm{rc}}$ as functions of pulling forces $F_{z}$ in Figure 7, where $F_{z}$ varies within the range from $0.5\;mr_{c}/\tau^{2}$ to $2.0\;mr_{c}/\tau^{2}$, with a small step of $0.25\;mr_{c}/\tau^{2}$. The rod–coil conformational transition period $t_{\mathrm{rc}}$ is obtained from the time-dependent shape factors shown in Figure 6 and is labeled accordingly. Here, the approximate values of $t_{\mathrm{rc}}$ with mean errors were obtained by average three replications. The results showed an exponential decrease in $t_{\mathrm{rc}}$ with increasing pulling force. The transition periods decrease as the pulling force increases because a strong pulling force more easily pulls the polymer chain into a rod-like conformation and then relaxes it to a coil conformation. The exponential decrease in transition periods provides more detailed information about the rod–coil conformational transition. However, other factors, such as the flexibility of the polymer chains and the solution environment can affect these conformational transitions [43].

#### 2.2.2. Conformations of Polymer Chains under Strong Adsorption

Strong adsorption probably introduces different types of information into the pulling processes because the strength of adsorption is one of the factors that affects the morphological changes of polymer chains [44]. We investigated the dynamic processes using various pulling forces when the interactions between the polymer chains and membranes are strong, specifically with $a_{{}_{\mathrm{PH}}}=-20$, as shown in Figure 8. The pulling forces chosen were $F_{z}=0.5\;mr_{c}/\tau^{2},1.0\;mr_{c}/\tau^{2},1.5\;mr_{c}/\tau^{2}$, and $2.0\;mr_{c}/\tau^{2}$ along the *z*-direction, that is, perpendicular to the membrane surfaces. For $F_{z}=0.5\;mr_{c}/\tau^{2}$, the shape factor first decreases and then increases, as shown in Figure 8a. At the end, $\langle \delta \rangle$ is less than 0.4, indicating that the polymer chains eventually assume a nearly spherical shape during the interaction with the membrane. Here, similar to the weak adsorption case where $F_{z}=0.5\;mr_{c}/\tau^{2}$, the polymer conformation obviously deviates from the coil conformation during the most of dynamics process in the strong adsorption case. At $F_{z}=1.0\;mr_{c}/\tau^{2}$, the $\langle \delta \rangle$ of the polymer chain undergoes an initial peak and becomes rod-like, followed by a gradual decrease with slight fluctuation. The $\langle \delta \rangle$ value again reaches 1, representing a rod conformation once again. This phenomenon is similar to the previous results and can be attributed to the damping effect, where the response gradually smoothens out over time under the influence of an external force [45]. In the case of $F_{z}=1.5\;mr_{c}/\tau^{2}$, the shape factor $\langle \delta \rangle$ exhibits two peaks under strong adsorption, indicating that the shape of the polymer chain undergoes rod-like conformations at two different moments during the relaxation process, as shown in Figure 8c. When $F_{z}=2.0\;mr_{c}/\tau^{2}$, the shape factor $\langle \delta \rangle$ partial trends similarly to $F_{z}=1.5\;mr_{c}/\tau^{2}$, as shown in Figure 8d, where the polymer chains also undergo a relaxation process, but there is an obvious difference that the behavior of $\langle \delta \rangle$ does not decrease again when it reaches a second peak, which is due to the limited time step and simulation box setting, and due to the fact that the whole motion process is always in the non-equilibrium state, the polymer chains change in a periodic motion, so we can observe the final return of the polymer chains to spherical shape at $F_{z}=1.5\;mr_{c}/\tau^{2}$. We observed that when the pulling forces are strong, the dynamic processes in the strong adsorption case ($a_{{}_{\mathrm{PH}}}=-20$) are similar to those in the weak adsorption case ($a_{{}_{\mathrm{PH}}}=-5$). However, this similarity does not hold when the pulling forces are weak. An important characteristic of the conformational transition is that the polymer chains undergo rod-like conformation twice, as shown in Figure 8, with the rod being perpendicular to the membrane surface during the first transition and parallel to the membrane surface during the second transition. This phenomenon has also been observed in weak adsorption cases. The parallel distribution of rods differs from that of polymer cylinders in electric fields, where the cylinders are oriented along the direction of the electric fields [46,47,48,49,50].

Similar to the cases in weak adsorption, we also obtained the relaxation periods in the strong adsorption cases, as shown in Figure 9. To observe the variances in the rod–coil conformational transition, we plotted the rod–coil transition periods $t_{\mathrm{rc}}$ as functions of pulling forces $F_{z}$, as shown in Figure 9. Here, $F_{z}$ varies from $0.5\;mr_{c}/\tau^{2}$ to $2.0\;mr_{c}/\tau^{2}$, with a small step of $0.25\;mr_{c}/\tau^{2}$. Similarly, the rod–coil transition period $t_{\mathrm{rc}}$ can be determined from the time-dependent shape factors shown in Figure 8. Here, the approximate values of $t_{\mathrm{rc}}$ with mean errors were obtained by average three replications. The results indicate that $t_{\mathrm{rc}}$ tends to decrease exponentially with increasing pulling force, which is consistent with observations in the weak adsorption cases. However, the difference lies in the fact that the polymer chain exhibits a larger $t_{\mathrm{rc}}$ in the strong adsorption case than in the weak adsorption cases when the pulling force is small. This can be attributed to the combined effect of weak pulling and strong adsorption. As the pulling force increases to a certain degree, this combined effect becomes weaker. In experiments, other factors, such as temperature and polymer chain concentration, can also affect conformational transitions in polymer chains [51]. In this study, we only focused on the influence of pulling force and adsorption between polymer chains and membrane surfaces. The pulling forces can be assumed in order of pN, which are the same order of forces in the single-molecule experiments [45].

#### 2.2.3. Order Parameters of Phospholipid Molecules

When the polymer chains are pulled away from the membrane surfaces, not only do conformational transitions occur in the polymer chains but the membrane can also be damaged. In this context, we used order parameters to describe the orientation of phospholipid molecules within the membranes. The order parameter can be expressed as [52,53]

(5) $$\langle P\left(\cos\theta \right)\rangle =\langle \frac{3}{2}\cos^{2}\theta -\frac{1}{2}\rangle ,\;$$

where θ is the angle between the polymer chain and the *z*-direction. The order parameter ranges from −0.5 to 1, where a value of 1 indicates that the polymer chain is parallel to the normal of the membrane surface (i.e., *z*-direction), whereas a value of −0.5 is perpendicular to the membrane surface when the order parameter is equal to −0.5. Intermediate values between 0 and 1 suggest partial alignment of the chain with the normal direction, and the chain is distributed randomly when the order parameter is equal to 0 [54].

Figure 10 depicts the order parameters for the hydrophilic heads of phospholipid molecules within the interaction zones with $x=20\;r_{c}∼60\;r_{c}$ and $y=20\;r_{c}∼60\;r_{c}$. Both weak and strong pulling forces and adsorption are considered, and the insets provide additional information. Figure 10a shows the order parameters for $F_{z}=0.5\;mr_{c}/\tau^{2}$ and $a_{{}_{\mathrm{PH}}}=-5$ at two pulling times, t=5τ and t=50τ, respectively. The results demonstrate that the fluctuations in the order parameter at t=50τ are higher than the initial t=5τ due to the effect of pulling forces. In the case of $F_{z}=2.0\;mr_{c}/\tau^{2}$, the detachment of the polymer chain from the surface occurs at the initial t=3τ, as shown in the inset of Figure 10b. This detachment leads to lower-order parameters for phospholipid molecules than with t=20τ. The strong fluctuations indicate that the polymer chains strongly affect the molecular arrangement on the membrane surfaces. Some differences are observed compared with the intermediate stage at $F_{z}=0.5\;mr_{c}/\tau^{2}$. This phenomenon occurs due to the increase in the pulling force, which leads to a faster movement of the polymer chains. The phospholipid beads in the upper layer are less affected by the movement of the polymer chains, resulting in less dramatic changes in the overall parameter. Next, we investigated the order parameters under strong adsorption, as shown in Figure 10c,d. The results showed that the order parameter of the phospholipid molecules changed in a similar manner when the polymer chains were pulled by weak and strong forces. This indicates that the damage area in the strong adsorption case is smaller than that in the weak adsorption case when the polymer chains are exfoliated from the phospholipid membranes, as shown in Figure 10c,d. This can be explained by the fact that strong adsorption provides a stronger binding force, allowing the polymer chains to bind more firmly to the phospholipid membrane and reducing damage. These observations suggest that the interaction between polymer chains and phospholipid membranes is more stable under strong adsorption, which helps to reduce both overall damage and localized damage during the detachment process. The presence or absence of the phospholipid membrane does not remarkably affect the order parameters. The rupture of the phospholipid membrane exhibits randomness and lacks obvious regularity, which is different from previous studies where pore formation was observed under tension in the experimental and theoretical sides [55,56,57,58].

## 3. Model and Method

### 3.1. Methodology

The DPD method is a CG simulation technique used for studying complex systems. In this method, molecules are divided into a collection of interacting beads, which are soft in nature [59,60]. Each bead represents a group of atoms or volumes of fluid that are relatively small at the macroscopic level but still large at the atomic scale. By employing appropriate force fields, DPD enables the connection between the microscopic and macroscopic levels, ensuring consistent length, time scales, and thermodynamic properties [61]. The DPD method has been widely used in soft-matter materials [62,63], hydrodynamics [64,65,66], and biofilm systems [67,68,69].

Here, we provide a brief description of the formulations used in the DPD simulation method. We denote a pair of randomly selected beads as *i* and *j* to illustrate the forces acting between them. Within this bead pair, three types of forces are found: the conservation force, the dissipative force, and the random force. The conservative force $\left(F_{ij}^{C}\right)$ accounts for the exclusion of volume effects between the beads. The dissipative force $\left(F_{ij}^{D}\right)$ represents the viscous resistance experienced by the beads. The random force $\left(F_{ij}^{R}\right)$ arises from the random forces exerted between the moving beads. To calculate the total force acting on the *i*-th bead, we sum up these forces, which can be expressed as

(6) $$F_{i}=\sum_{i\neq j}\left(F_{ij}^{C}+F_{ij}^{D}+F_{ij}^{R}\right)=\sum_{i\neq j}\left[a_{ij}w\left(r_{ij}\right)-\gamma w^{2}\left(r_{ij}\right)\left(r_{0}\cdot v_{ij}\right)+\sigma w\left(r_{ij}\right)\zeta_{ij}\Delta t^{-\frac{1}{2}}\right]r_{0},$$

where $a_{ij}$ is the maximum repulsive force between the *i*-th and *j*-th beads. The relative distance and relative velocity between the *i*-th and *j*-th beads are denoted by $r_{ij}$ (with the unit vector $r_{0}$) and $v_{ij}$, respectively. $\zeta_{ij}$ is a random variable with Gaussian distribution and unit variance [70]. Equation $\sigma^{2}=2\gamma k_{B}T$ relates the parameters γ and σ, where $k_{B}$ is the Boltzmann constant, γ=4.5, and σ=3.0 [67,71]. The coefficient $w\left(r_{ij}\right)$ denotes the normalized distribution function,

(7) $$w=\left\{\begin{array}{cc} 1-\frac{r_{ij}}{r_{c}} & r_{ij}<r_{c}\\ 0 & r_{ij}>r_{c} \end{array},\right.$$

where $r_{c}$ is the cut off radius.

### 3.2. Model

We developed a phospholipid membrane and macromolecular chain models based on a CG model. CG modeling involves averaging a set of nonessential degrees of freedom and grouping several atoms into a bead [72]. The CG force field is then adjusted to capture the fundamental properties of the system [73,74,75]. Figure 1 depicts the membranes and polymer chains, where (a) and (b) represent cases with pulling forces applied parallel and perpendicular to the membrane surfaces, respectively. The parameters for both the model membrane and polymer chains are the same. In the phospholipid membrane model, shown in Figure 1, we represent the hydrophilic and hydrophobic tail chains with pink and blue beads, respectively [76,77]. The membrane model consists of a head chain and two tail chains, where a group of atoms is treated as a single bead. Additionally, an elastic harmonic force is applied between consecutive beads, as illustrated in Figure 1, given by the equation

(8) $$F_{ij}=k_{s}\left(1-\frac{r_{ij}}{r_{s}}\right)r_{0}\;,\;$$

where $k_{s}$ and $r_{s}$ denote the spring constants and equilibrium bond lengths of consecutive beads, respectively. In this work, we set their values to $k_{s}=120.0$ and $r_{s}=0.7\;r_{c}$, similar to previous studies [30,78]. Another set of forces is applied to the two consecutive bonds

(9) $$F^{\theta }=-\nabla \left[k_{\theta }{\left(\theta -\theta_{0}\right)}^{2}\right]\;,\;$$

where $k_{\theta }=6$ represents the bending constant of the bond, θ=π represents the tilt angle between three consecutive head particles or tail particles, and $\theta_{0}=\frac{2}{3}\pi$ represents the equilibrium angle of the three consecutive beads at the junction point. We set the maximum repulsion between identical beads to $a_{ij}=25$ and between different particles in the membrane model to $a_{ij}=100$, consistent with previous studies [30,67]. In the simulations, we immobilized the phospholipid film on the aqueous substrate, fixing the bottom two head beads of the phospholipid.

**Figure 11:**
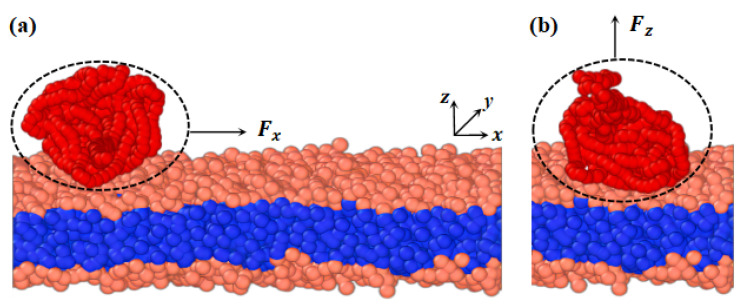
Schematic diagram of the phospholipid membrane and linear polymer model. The phospholipid membrane consists of one hydrophilic chain (pink head beads) and two hydrophobic chains (blue tail beads). The linear polymer (red beads) interacts with the hydrophilic head chains on the upper surface of the phospholipid membrane. (**a**) The pulling force applied to the polymer chain is along the *x*-direction. (**b**) The pulling force applied to the polymer chain is along the *z*-direction.

**Figure 12:**
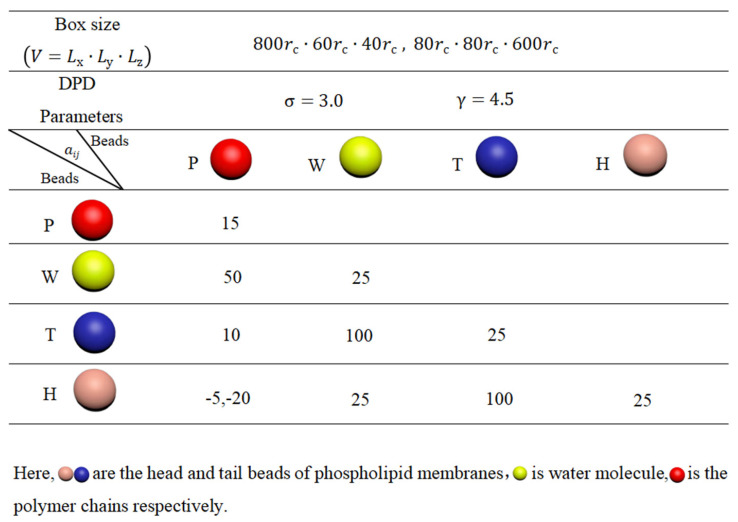
System parameters in the simulation box.

For the polymer chains, we set $k_{s}=200$ and $r_{s}=0.5\;r_{c}$ to maintain linear topology under different pulling forces. The length of the linear chain is N=500. The repulsion parameters are set to $a_{ij}=15$ for the beads of the same type and $a_{ij}=50$ for different types of polymer beads, represented in red in Figure 11. These interaction parameters, related to the Flory–Huggins interaction parameter χ, allow us to observe the effect of pulling [79]. Figure 12 summarizes the interaction parameters between the DPD beads, where P denotes the polymer bead, W represents the water bead, and T and H indicate the tail and head beads in phospholipid chains, respectively. All these various types of beads are labeled with different colors. The DPD parameters used in this study are similar to those employed in previous research [77,80,81,82].

### 3.3. Simulation Parameters

In the current simulations, the energy is normalized by $k_{B}T$, and the length is normalized by the cut off radius $r_{c}$. The cut off radius is determined by the bead density ρ=3 and volume $V_{b}$, such that $r_{c}={\left(\rho V_{b}\right)}^{1/3}$. We set ρ=3 for the particle density, and the cut off radius is chosen as $r_{c}=0.5$ nm, consistent with previous studies [30,35,83]. The time is normalized by τ, which is calculated as follows:

(10) $$\tau =r_{c}\sqrt{\frac{m}{k_{B}T}}\;,\;$$

where *m* represents the mass of the bead, which is taken as the unit mass in the simulations. We adopted a modified version of the *velocity-Verelt* algorithm, with a time step Δt=0.005τ. All of the simulations were performed using the NVT ensemble in the LAMMPS simulator [77,84]. In the case of parallel pulling, the simulation box size was set to $800\;r_{c}\times 60\;r_{c}\times 40\;r_{c}$. In the case of perpendicular pulling, the simulation box size was set to $80\;r_{c}\times 80\;r_{c}\times 600\;r_{c}$. Periodic boundary conditions were applied in all three directions in both cases, and the box size was chosen to be sufficiently large along the pulling direction to observe the pulling effects within a single pulling process. In the first case, the linear polymer chains are pulled from left to right along the membrane surface, while in the second case the chains are pulled from the membrane surface to the top of the simulation box. Setting $L_{{}_{x}}=800\;r_{c}$ and $L_{{}_{z}}=600\;r_{c}$ in both cases makes the pulling time of the polymer chains relatively long, which allows us to observe the dynamics processes in more detail. Throughout the pulling process, the force is always applied to the polymer chains, which results in a non-equilibrium state and varies periodically in our observation range. In both cases, the linear polymer chains were randomly distributed on the membrane surface. We performed the pulling simulations at least three times for the identical system parameters by inputting different random initial conformations of linear polymer chains.

In the current simulations, we set $a_{{}_{\mathrm{PH}}}=-5$ as the weak adsorption and $a_{{}_{\mathrm{PH}}}=-20$ as the strong adsorption. This is a relative definition according to the adsorption effects of polymer chains on the membranes. Namely, the strong adsorption leads to more obvious damage of the phospholipid membrane than those in the weak adsorption case. The similar definition has been also used in the previous experiment and DPD simulation [35,85]. Actually, the interaction parameters are related to the Flory–Huggins interaction parameters, which are determined by the physical and chemical properties of materials. Subsequently, we applied pulling forces **F** to each bead of the polymer chains, that is, along the *x* direction in the parallel pulling case and the *z* direction in the perpendicular pulling case. These pulling forces can be implemented to mimic the effect of an external electric field in electrolyte experiments [86,87]. It is worthwhile to relate the force unit $\;mr_{c}/\tau^{2}$ to the unit commonly used in the experiments. Here, we consider a 5000 bp dsDNA chain with the total mass of $M\approx 5.3\times 10^{-21}\;\mathrm{kg}$ and then coarse-grain this dsDNA sequence into a chain with *N* DPD beads, where N=500. Thus, one DPD bead has a mass of $m\approx 1.06\times 10^{-23}\;\mathrm{kg}$ since the time unit τ and length unit $r_{c}$ are approximately τ=1.88ns and $r_{c}=0.5\;\mathrm{nm}$ [71]. This yeilds $mr_{c}/\tau^{2\;}=1.3\times 10^{-3}\;\mathrm{pN}$. Therefore, the total pulling forces acting on the whole polymer chain with 500 DPD beads (5000 bp dsDNA) can be expressed as $F=NF_{x}=500\times 2.0\;\;mr_{c}/\tau^{2\;}=1.3\;\mathrm{pN}$ when $F_{x}=2.0\;\;mr_{c}/\tau^{2\;}$. This is the same order of magnitude as the pulling force acting on a dsDNA chain in single-molecule experiments [45,88].

## 4. Conclusions

In this study, we performed DPD simulations using CG models to investigate the interaction between linear polymer chains and phospholipid membranes in solution. We developed two models where external forces were applied to the linear chains in parallel and perpendicular directions to the membrane surfaces. We used the gyration radius and shape factor to examine the conformational transitions of polymer chains and employed the bead number and order parameters to observe the properties of membranes during the dynamic processes. We considered weak and strong adsorptions, specifically $a_{{}_{\mathrm{PH}}}=-5$ and −20, along with various pulling forces $F=0.5\;mr_{c}/\tau^{2},1.0\;mr_{c}/\tau^{2},1.5\;mr_{c}/\tau^{2}$, and $2.0\;mr_{c}/\tau^{2}$ in both cases.

For weak adsorption in the parallel pulling case, the gyration radius and shape factor indicated that the polymer chain undergoes a coil–circle conformational transition under weak pulling forces of $F_{x}=0.5\;mr_{c}/\tau^{2}$ and $1.0\;mr_{c}/\tau^{2}$, whereas it undergoes a coil–rod conformational transition under strong pulling forces of $F_{x}=1.5\;mr_{c}/\tau^{2}$ and $2.0\;mr_{c}/\tau^{2}$. For strong adsorption in the parallel pulling mode, similar coil–rod conformational transitions were observed but more prominently than in the weak adsorption cases. The shape factor exhibited similar oscillatory periods when $F_{x}=0.5\;mr_{c}/\tau^{2}$ and $1.5mr_{c}/\tau^{2}$, respectively, but not when $F_{x}=1.0\;mr_{c}/\tau^{2}$ and $2.0\;mr_{c}/\tau^{2}$. We observed that the oscillation periods are longer in strong adsorption than in weak adsorption due to the interaction between polymer chains and membranes, which can be explained by the spring model. The membranes were also affected by the pulling polymer chains. The bead number $n_{{}_{H}}$ indicated that more lipid molecules were detached from the membrane surfaces under smaller pulling forces than with stronger pulling forces in the case of weak adsorption in the parallel pulling case. Moreover, the results showed that the phospholipid membrane can undergo rapid repair under strong pulling forces. In the case of strong adsorption, a higher number of molecules can detach from the membrane surfaces due to the stronger interactions between the polymers and membranes.

In the case of weak adsorption with the perpendicular pulling case, we also observed rod–coil conformational transitions under pulling forces. The polymer chain can quickly transition into rod-like conformations under strong pulling forces. Subsequently, fluctuations in the shape factor occur as the polymer chains are pulled away from the membranes. The pulling time *t* within the simulation boxes decreases as the pulling force increases. In the case of strong adsorption with perpendicular pulling, the polymer chain also undergoes rod–coil conformational transitions. However, the polymer chain exhibits different rod conformations during the dynamic process, initially perpendicular to the membrane surface and eventually parallel to the membrane surface. Another characteristic is the exponential decrease in the rod–coil conformational transition periods $t_{\mathrm{rc}}$, with increasing pulling forces $F_{z}$ in both weak and strong adsorption cases. Regarding the membrane features during the pulling processes, the results showed that fluctuations in the order parameter became stronger due to the effect of pulling forces $F_{z}$, indicating that the polymer chains strongly affected the arrangement of molecules on the membrane surfaces. The results also suggested that the detachment of phospholipid molecules results in lower ordering of the phospholipid molecules. The order parameters of the phospholipid molecules are similar when the polymer chains are pulled by weak and strong forces, despite the stronger binding force provided by strong adsorption. These parameters were discussed via comparison with the other simulation and experimental observations. The simulation results can further deepen the understanding of the interaction mechanism between the polymer membrane and phospholipid membrane and have potential applications in biomedicine.

## Data Availability

Not applicable.

## Supplementary Materials

The following supporting information can be downloaded at: https://www.mdpi.com/article/10.3390/molecules28155790/s1. Figures S1–S8: Replicas of dynamics processes.
