# Supplementary material for: Interaction of Macromolecular Chain with Phospholipid Membranes in Solutions: A Dissipative Particle Dynamics Simulation Study
Source: Molecules. 2023 Jul 31;28(15):5790. doi: 10.3390/molecules28155790 (PMC10420874; doi:10.3390/molecules28155790)
Supplement: Supplementary file 1 [file molecules-28-05790-s001.zip › molecules-2513734-supplementary.pdf]

Supplementary Materials

Interaction of Macromolecular Chain with Phospholipid Membranes in Solutions: A dissipative particle dynamics simulation study

Yuane Wang [1], Xuankang Mou [1] , Yongyun Ji [1], Fan Pan [2*] and Shiben Li [1*]

1 Department of Physics, Wenzhou University, Wenzhou, Zhejiang 325035, China.

2 School of Data Science and Artificial Intelligence, Wenzhou University of Technology, Wenzhou, Zhejiang 325035, China.

*Correspondence: panfan@wzu.edu.cn (F. P.); shibenli@wzu.edu.cn (S. L.)

Here, we provide three replicas of dynamics processes for each set of parameters used in the DPD simulations.

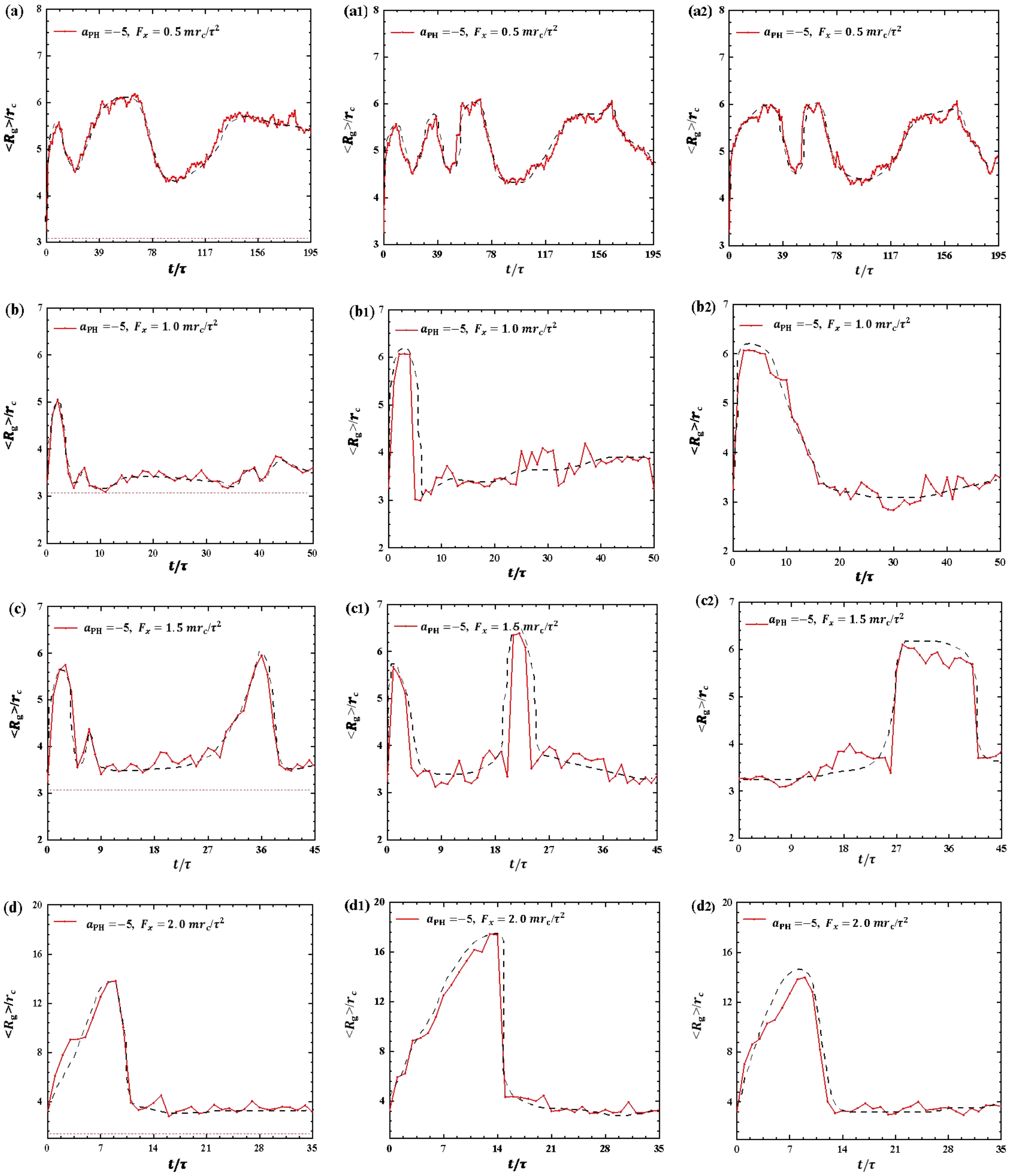

Figure S1. Variation of gyration radius $\langle R_g \rangle$ for the polymer chains in the weak adsorption cases of $a_{PH} =- 5$. The pulling forces applied to the polymer chains are along the x direction for $F_x = 0.5\ mr_c/\tau^2$ [(a), (a1) and (a2)], $F_x = 1.0\ mr_c/\tau^2$ [(b), (b1) and (b2) are three replications], $F_x = 1.5\ mr_c/\tau^2$ [(c), (c1) and (c2)], and $F_x = 2.0\ mr_c/\tau^2$ [(d), (d1) and (d2)]. The dashed fitting lines show the variation trends. The letters in square brackets correspond to three replicas at the identical system parameters with various random inputting.

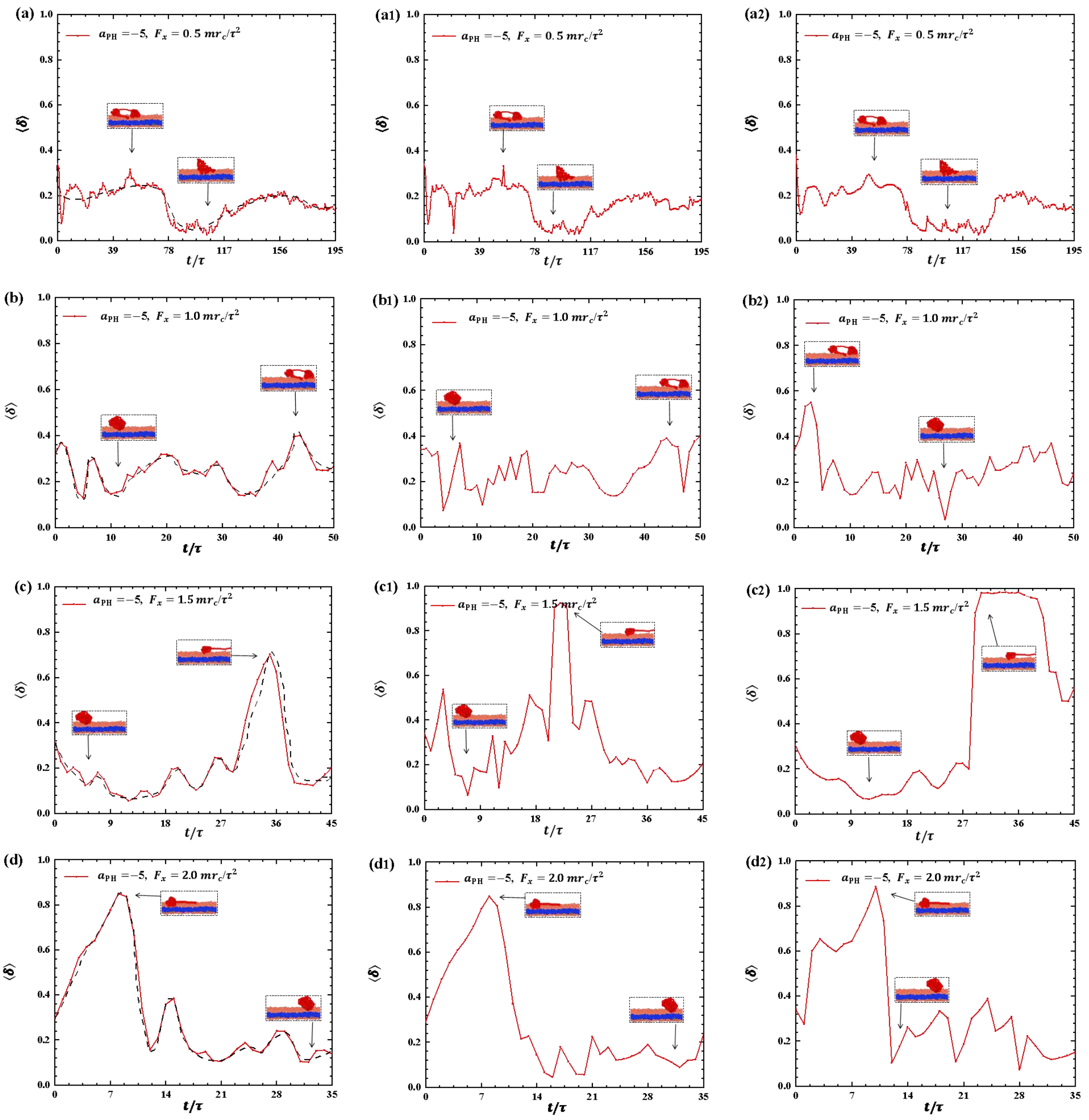

Figure S2. Variation of shape factor $\langle\delta\rangle$ for the polymer chains in the weak adsorption cases of $a_{\mathrm{PH}} =-5$. The pulling forces applied to the polymer chains are along the x direction for $F_x = 0.5\ mr_{\mathrm{c}}/\tau^2$ [(a), (a1) and (a2)], $F_x = 1.0\ mr_{\mathrm{c}}/\tau^2$ [(b), (b1) and (b2)], $F_x = 1.5\ mr_{\mathrm{c}}/\tau^2$ [(c), (c1) and (c2)], and $F_x = 2.0\ mr_{\mathrm{c}}/\tau^2$ [(d), (d1) and (d2)]. The dashed fitting lines show the variation trends and the typical conformations are also inserted. The letters in square brackets correspond to three replicas at the identical system parameters with various random inputting.

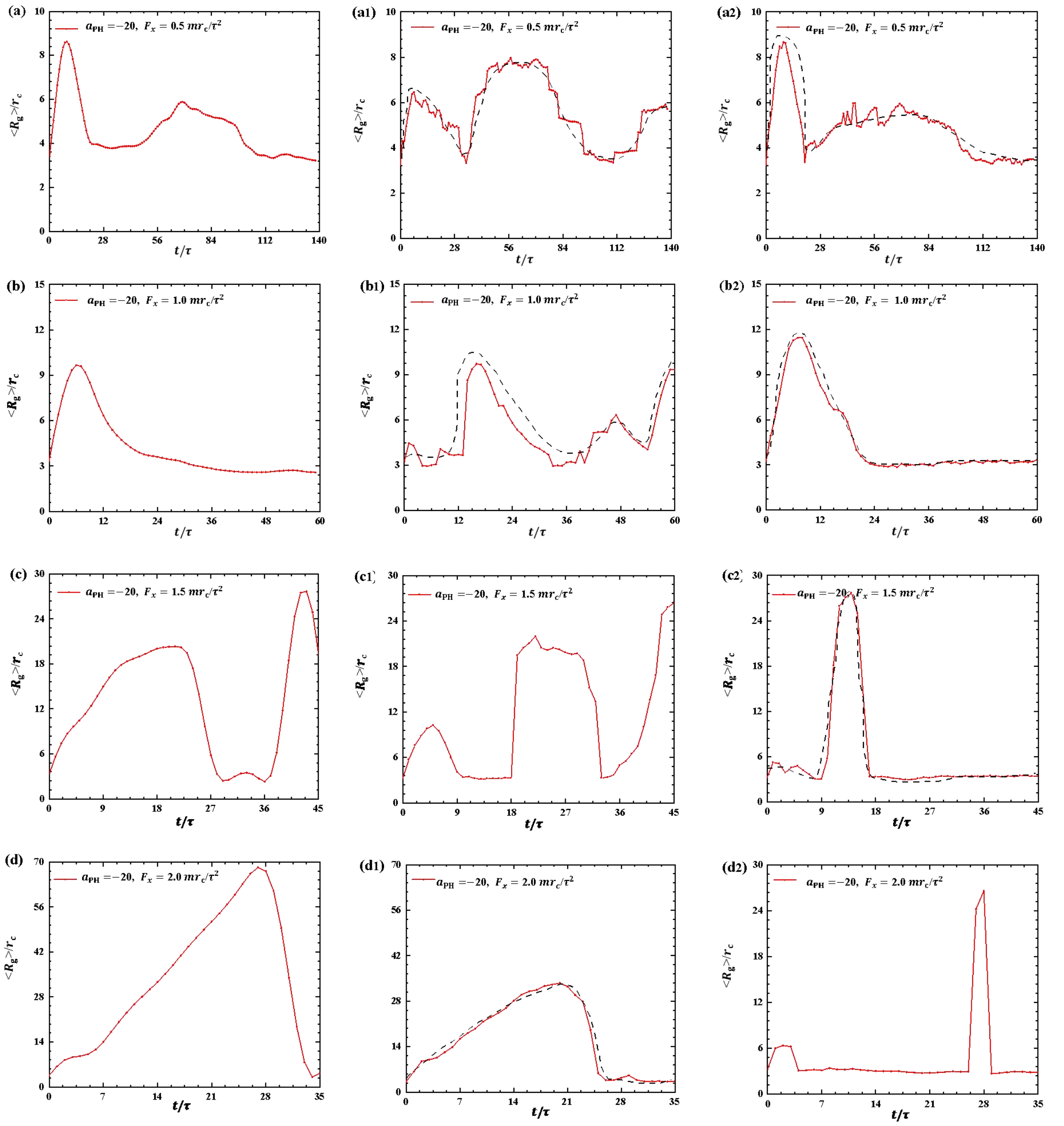

Figure S3. Variation of gyration radius $\langle R_{\rm g}\rangle$ for the polymer chains in the weak adsorption cases of $a_{\rm PH} =-20$. The pulling forces applied to the polymer chains are along the x direction for $F_x = 0.5\ mr_{\rm c}/\tau^2$ [(a), (a1) and (a2)], $F_x = 1.0\ mr_{\rm c}/\tau^2$ [(b), (b1) and (b2) are three replications], $F_x = 1.5\ mr_{\rm c}/\tau^2$ [(c), (c1) and (c2)], and $F_x = 2.0\ mr_{\rm c}/\tau^2$ [(d), (d1) and (d2)]. The dashed fitting lines show the variation trends. The letters in square brackets correspond to three replicas at the identical system parameters with various random inputting.

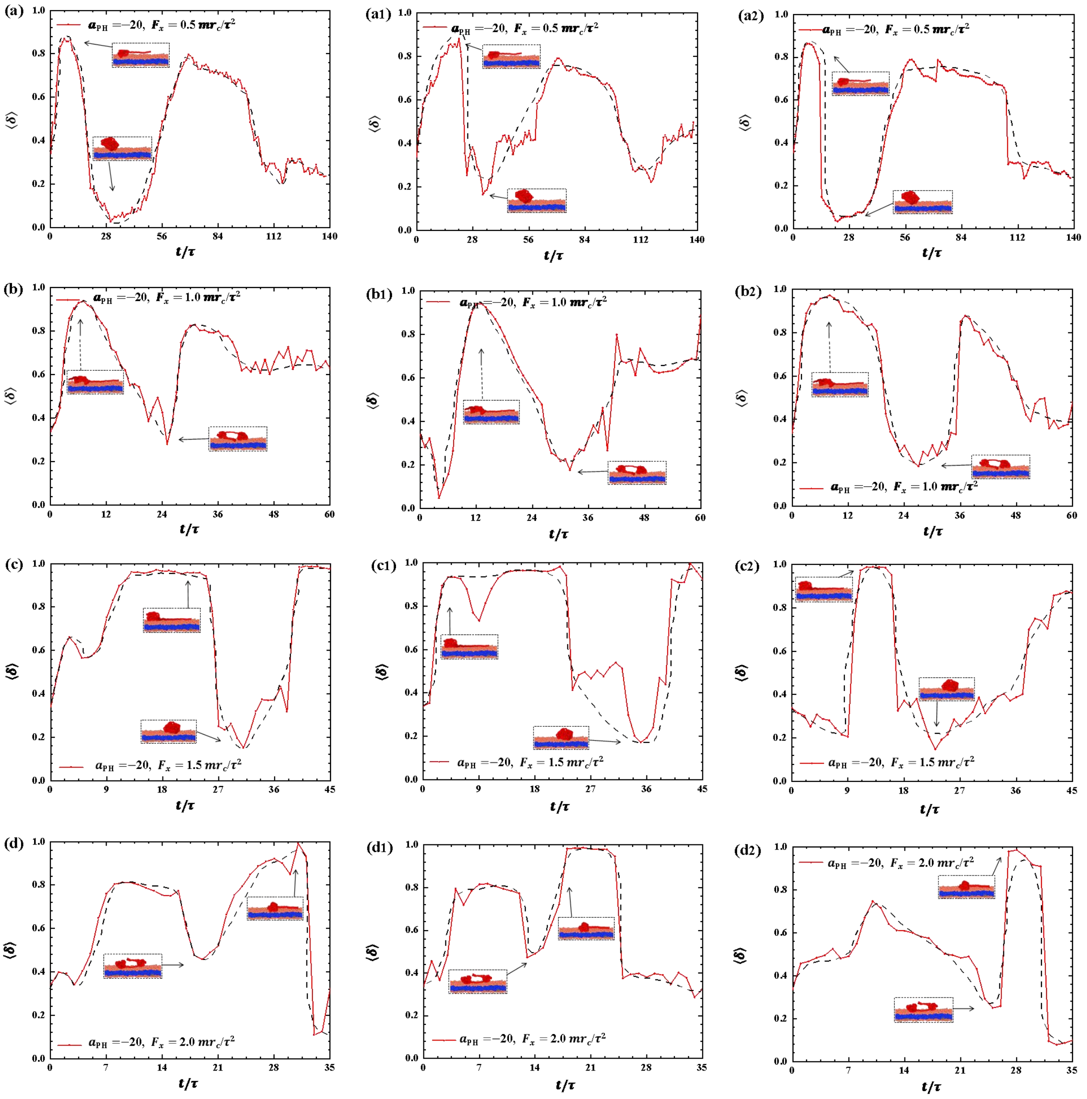

Figure S4. Variation of shape factor $\langle\delta\rangle$ for the polymer chains in the weak adsorption cases of $a_{\mathrm{PH}} =-20$. The pulling forces applied to the polymer chains are along the x direction for $F_x = 0.5\ mr_{\mathrm{c}}/\tau^2$ [(a), (a1) and (a2)], $F_x = 1.0\ mr_{\mathrm{c}}/\tau^2$ [(b), (b1) and (b2) are three replications], $F_x = 1.5\ mr_{\mathrm{c}}/\tau^2$ [(c), (c1) and (c2)], and $F_x = 2.0\ mr_{\mathrm{c}}/\tau^2$ [(d), (d1) and (d2)]. The dashed fitting lines show the variation trends, and the typical conformations are also inserted. The letters in square brackets correspond to three replicas at the identical system parameters with various random inputting.

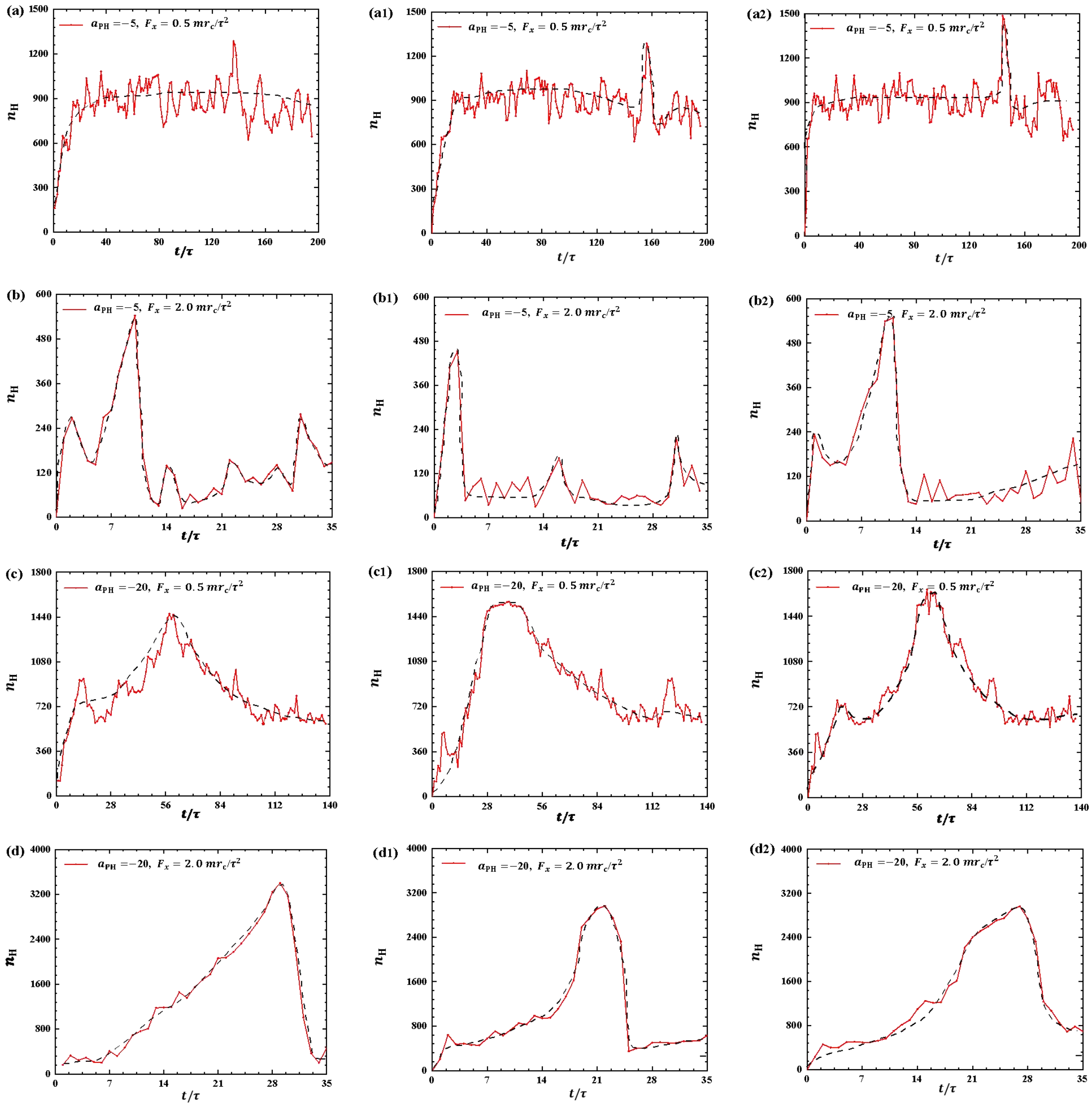

Figure S5. The number of hydrophilic phospholipid molecules lie in the range $z = 10\,r_c - 15\,r_c$ as functions of the pulling time in the cases of [(a), (a1) and (a2) are three replications] weak adsorption $a_{PH} =- 5$ and weak pulling force $F_x = 0.5\,mr_c/\tau^2$, [(b), (b1) and (b2)] weak adsorption $a_{PH} =- 5$ and strong pulling force $F_x = 2.0\,mr_c/\tau^2$, [(c), (c1) and (c2) are three replications] strong adsorption $a_{PH} =- 20$ and weak pulling force $F_x = 0.5\,mr_c/\tau^2$ and [(d), (d1) and (d2) are three replications] strong adsorption $a_{PH} =- 20$ and strong pulling force $F_x = 2.0\,mr_c/\tau^2$. The letters in square brackets correspond to three replicas at the identical system parameters with various random inputting.

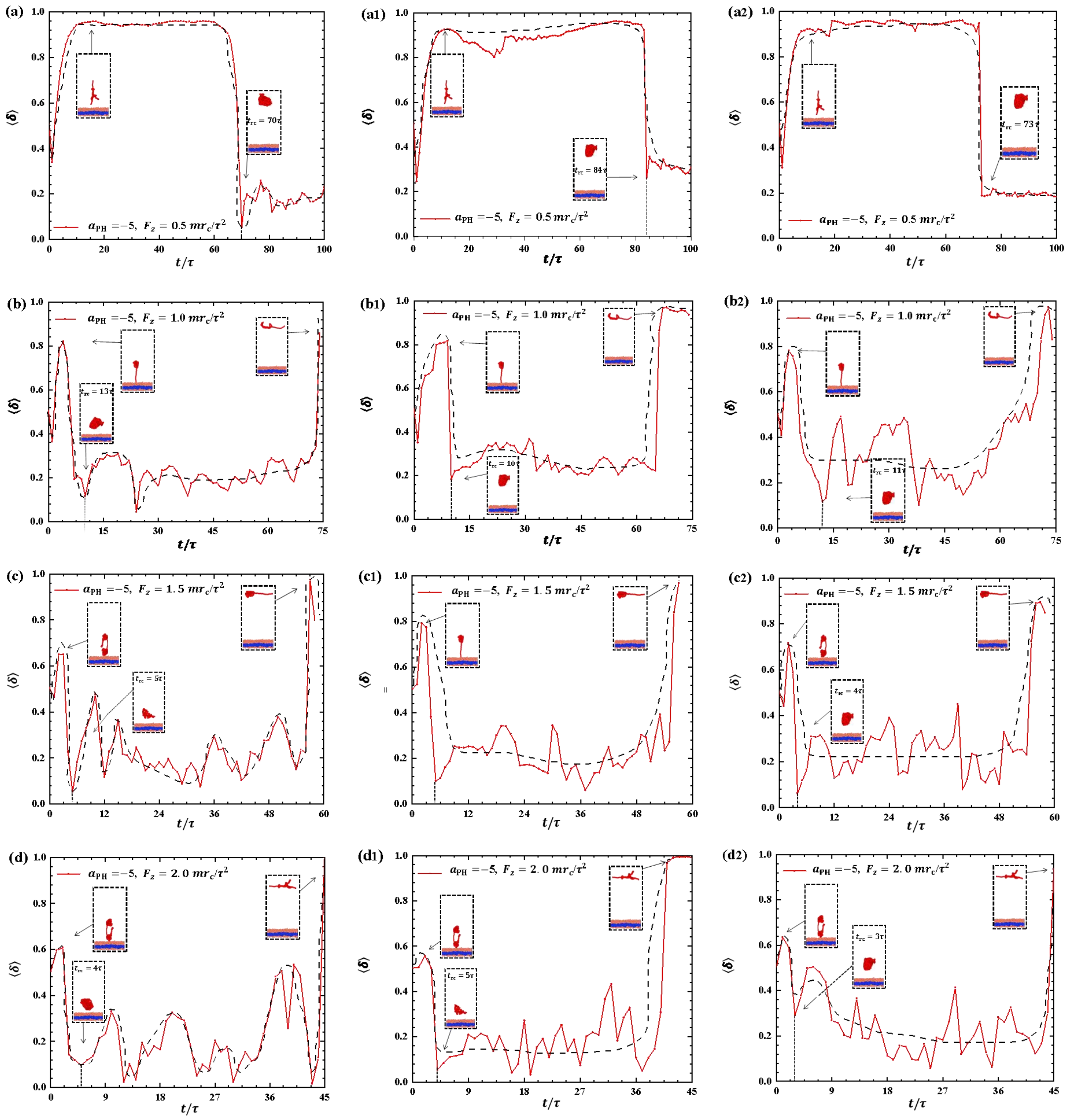

Figure S6. Variation of shape factor $\langle\delta\rangle$ for the polymer chains in the weak adsorption cases of $a_{\mathrm{PH}} =- 5$. The pulling forces applied to the polymer chains are along the z direction for $F_z = 0.5\ mr_{\mathrm{c}}/\tau^2$ [(a), (a1) and (a2)], $F_z = 1.0\ mr_{\mathrm{c}}/\tau^2$ [(b), (b1) and (b2) are three replications], $F_z = 1.5\ mr_{\mathrm{c}}/\tau^2$ [(c), (c1) and (c2)], and $F_z = 2.0\ mr_{\mathrm{c}}/\tau^2$ [(d), (d1) and (d2)]. The dashed fitting lines show the variation trends and the typical conformations are also inserted. The letters in square brackets correspond to three replicas at the identical system parameters with various random inputting.

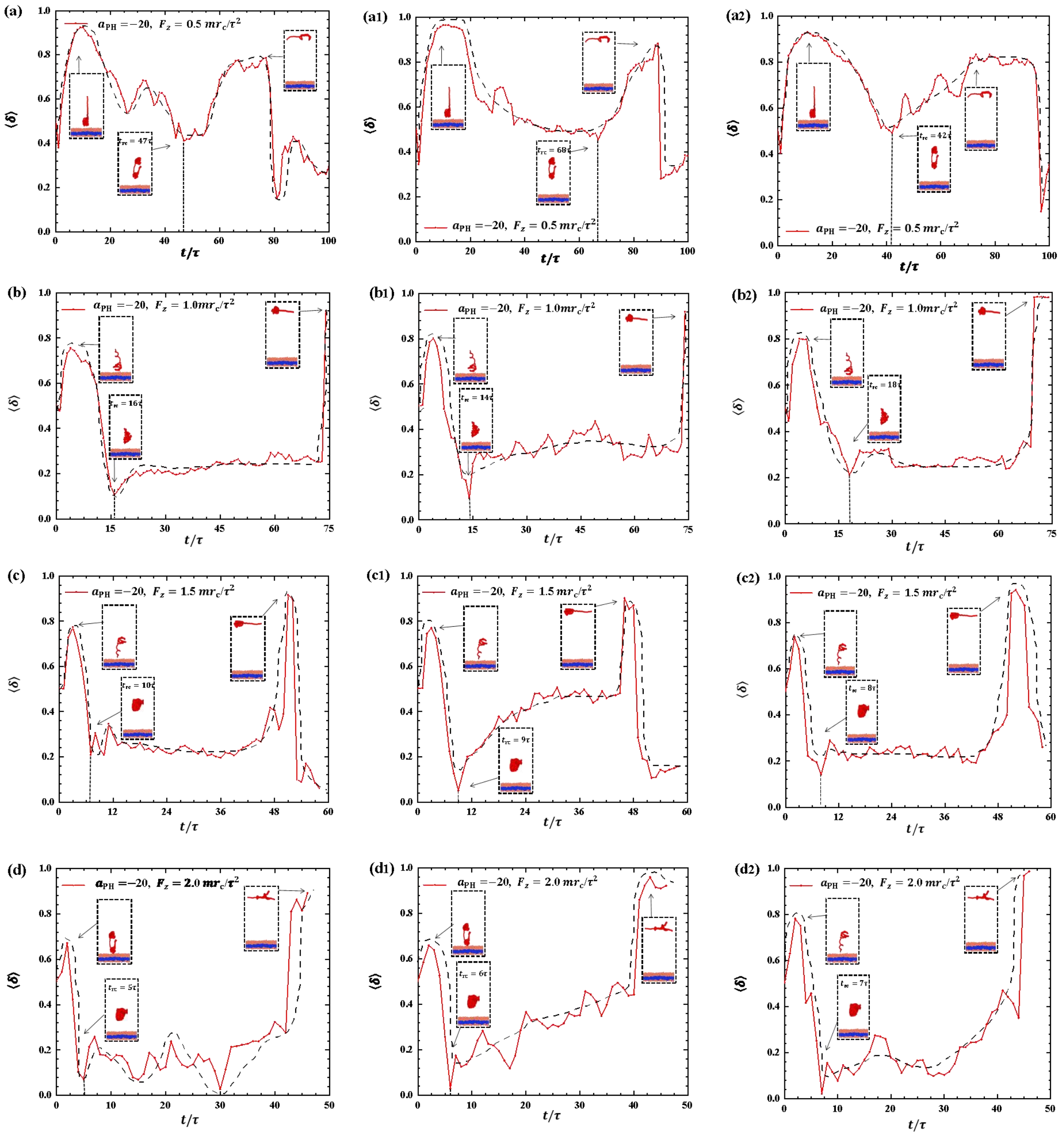

Figure S7. Variation of shape factor $\langle\delta\rangle$ for the polymer chains in the weak adsorption cases of $a_{\mathrm{PH}} =-20$. The pulling forces applied to the polymer chains are along the z direction for $F_z = 0.5\ mr_{\mathrm{c}}/\tau^2$ [(a), (a1) and (a2)], $F_z = 1.0\ mr_{\mathrm{c}}/\tau^2$ [(b), (b1) and (b2) are three replications], $F_z = 1.5\ mr_{\mathrm{c}}/\tau^2$ [(c), (c1) and (c2)], and $F_z = 2.0 mr_{\mathrm{c}}/\tau^2$ [(d), (d1) and (d2)]. The dashed fitting lines show the variation trends, and the typical conformations are also inserted. The letters in square brackets correspond to three replicas at the identical system parameters with various random inputting.

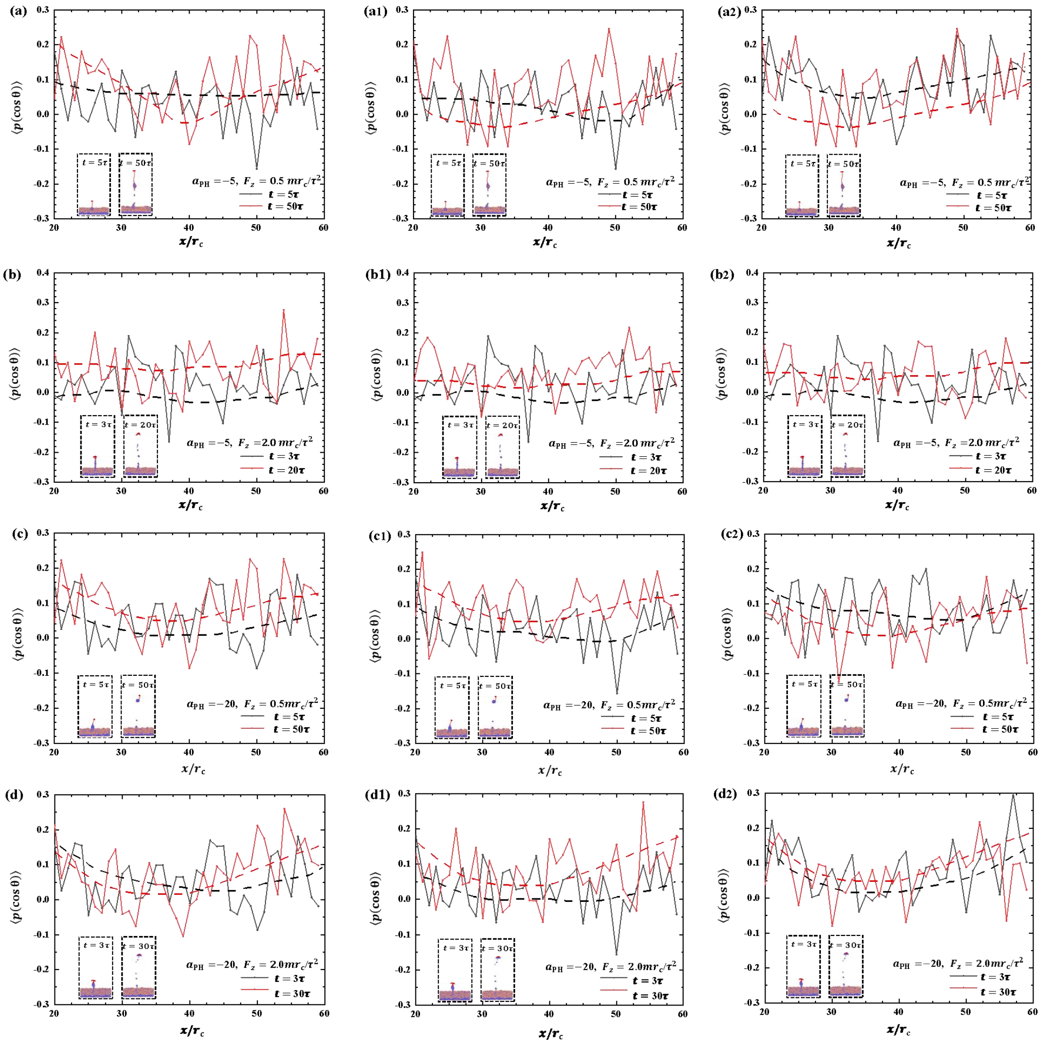

Figure S8. Order parameter profiles of phospholipid films in the cases of [(a), (a1) and (a2) are three replications] weak adsorption $a_{\mathrm{PH}} =- 5$ and weak pulling force $F_z = 0.5\ mr_{\mathrm{c}}/\tau^2$, [(b), (b1) and (b2)] weak adsorption $a_{\mathrm{PH}} =- 5$ and strong pulling force $F_z = 2.0\ mr_{\mathrm{c}}/\tau^2$, [(c), (c1) and (c2)]strong adsorption $a_{\mathrm{PH}} =- 20$ and weak pulling force $F_z = 0.5\ mr_{\mathrm{c}}/\tau^2$ and [(d), (d1) and (d2)]strong adsorption $a_{\mathrm{PH}} =- 20$ and strong pulling force $F_z = 2.0\ mr_{\mathrm{c}}/\tau^2$. The letters in square brackets correspond to three replicas at the identical system parameters with various random inputting.
